# Dysregulation of microRNA (miRNA) Due to Phthalate/Phthalate Metabolite Exposure and Associated Health Effects: A Narrative Review

**DOI:** 10.3390/jox15030072

**Published:** 2025-05-12

**Authors:** Aamer Mohammed, Stephen L. Atkin, Edwina Brennan

**Affiliations:** 1 School of Medicine, Royal College of Surgeons in Ireland Bahrain, Adliya 15503, Bahrain; 21200392@rcsi-mub.com; 2 School of Postgraduate Studies & Research, Royal College of Surgeons in Ireland Bahrain, Adliya 15503, Bahrain; satkin@rcsi.com

**Keywords:** endocrine disrupting chemicals (EDCs), plasticisers, phthalates, phthalate metabolites, epigenetics, microRNA (miRNA)

## Abstract

Phthalates, a group of synthetic non-persistent organic chemicals commonly used as solvents and plasticisers, have been associated with a range of detrimental health effects. These endocrine disrupting chemicals (ECDs) may exert their effects through epigenetic changes such as altered microRNA (miRNA) expression. miRNAs are short non-coding endogenous RNA transcripts that are preferentially expressed in various tissues and cell types and can circulate in body fluids, thereby regulating gene expression and acting as mediators for intercellular communication. As miRNAs mostly target protein-coding transcripts, they are involved in nearly all networks that regulate developmental and pathological processes. In this review, we provide an overview of human, in vivo and in vitro studies assessing altered miRNA expression due to phthalate exposure and their biological effects. Importantly, this study suggests that the mechanism of phthalate action may in part be mediated by epigenetic changes, affecting a large number of different proteins. This is indicative that alterations in miRNA expression induced by phthalate exposure are then implicated in a wide range of health conditions, including reproductive dysfunction, oncogenesis, metabolic disorders, and neurodevelopmental outcomes. Exposure to phthalates and their metabolites predominantly results in the upregulation of miRNAs. Dysregulation of miR-34a, miR-15b, miR-141, miR-184, miR-19a, miR-125, and miR-let-7 were observed across several studies. More research involving human participants combined with mechanistic studies integrating mRNA target analysis would be beneficial in understanding the downstream effects of phthalate exposure on gene expression and grasping the broader biological implications.

## 1. Introduction

Phthalates, diesters of phthalic acid (1,2-benzenedicarboxylic acid), are a group of synthetic organic chemicals commonly used as solvents and plasticisers in industrial and commercial products. Phthalate manufacturing began in the 1920s [1] with an estimated global production of 6 million tonnes in 2017, having more than doubled compared to the previous decade [2,3]. Food packaging, personal care products (PCPs) (cosmetics, perfumes, hair care, soaps), childcare articles (toys, teethers), medical devices, and building materials (paints, floorings) invariably contain phthalates [4]. Not being chemically bound to polymers, phthalates have the ability to leach, reportedly at levels above regulator-allowed limits [5], resulting in exposure due to skin contact, inhalation, and, predominantly, ingestion [4]. Consequently, phthalates are ubiquitous in environmental matrices [6] and are commonly detected in body fluids (urine, blood, seminal fluid, breast milk, amniotic fluid) and tissues in human biomonitoring studies [4,7].

Low-molecular-weight phthalates (LMWPs) are classified as those with ester-side chain lengths of one to four carbons and high-molecular-weight phthalates (HMWPs) as five or more carbons [8] (Table 1). LMWPs are primarily used as solvents in various consumer and PCPs, while HWMPs are generally used as additives in polyvinyl chloride (PVC) products [9]. In the body, phthalates are rapidly metabolised with half-lives on the order of hours [10,11] via a two-phase process; an initial enzymatic (esterase or lipase) hydrolysis of the parent diester to its primary monoester metabolite, whose side chain may undergo hydroxylation or oxidation, followed by uridine 59-diphosphoglucuronyl transferase glucuronidation prior to excretion, primarily in urine [12].

Phthalates are known endocrine disruptors with the ability to interfere with the production, release, and/or function of hormones [14]. Recent publications provide strong evidence for the reproductive, neurodevelopmental, cardiovascular, metabolic, and carcinogenic toxicity of phthalate exposure [7,15,16], which has led to restrictions on the use of phthalates in Japan, China, the US, and Europe. In Europe, for example, levels of di-2-ethylhexyl-phthalate (DEHP), di-n-butyl phthalate (DBP), benzyl butyl phthalate (BzBP), and di-isobutyl phthalate (DiBP) at ≥0.1% by weight are banned in certain plastic articles [17].

In recent years, greater emphasis has been placed on the role of epigenetics, i.e., altered gene activity due to DNA methylation, histone modification, and non-coding RNA action, in the development of disease. MicroRNAs (miRNAs) are short non-coding endogenous RNA transcripts measuring ~22 nucleotides long that alter gene expression post-transcriptionally [18]. There are two classes of miRNA, those encoded from overlapping introns of protein coding regions and those from exons [19]. miRNA transcripts are initially transcribed in the nucleus by RNA polymerases to yield primary (pri)-miRNA, consisting of a step–loop structure [20,21]. In the canonical pathway, subsequent cleavage of pri-miRNA by the microprocessor complex yields the hairpin precursor (pre)-miRNA, which is exported to the cytoplasm via Ran-dependent nuclear transport receptor protein. In the cytoplasm, further processing by DICER gives rise to a mature miRNA duplex, the guide strand of which is loaded into the argonaute family of proteins to form an miRNA-induced silencing complex (miRISC) [20,21]. Other less common pathways for miRNA biogenesis have been reported independent of DROSHA, a component of the microprocessor complex [22], and DICER [23]. The subsequent miRNA binds via complementary binding to the 3′ untranslated regions of target mRNAs, resulting in either the inhibition of target translation or the promotion of target degradation [24]. Similar levels of inhibition are also reported via binding to 5′ untranslated region [25], while induced expression via promoter binding has been proposed [26]. miRNAs are known to be expressed differently in various tissues and cell types [27], can be localised in various cellular compartments (e.g., nucleus, rough endoplasmic reticulum, mitochondria) to promote efficient gene regulation [21], and can circulate in body fluids within extracellular vesicles (EV-miRNA) or bound to protein, thereby acting as mediators for intercellular communication [28]. Although the sorting of miRNA in exosomes is not fully understood, argonaute proteins and other RNA-binding proteins are thought to play a role [28]; meanwhile, the release of exosomal miRNA has been proposed via a ceramide-dependent pathway [29]. Given the short nucleotide sequence, a single miRNA may be complementary with hundreds of mRNA 3′ untranslated regions; a single mRNA may be influenced by many miRNAs [30]. Consequently, miRNAs are involved in nearly all networks that regulate developmental and pathological processes and are potentially promising biomarkers for disease and diagnostics [27].

Recent reviews have focused on exposure to environmental pollutants and dysregulation of miRNAs in an effort to assess miRNA consistency and the direction of their associations and roles in the pathophysiology of disease [31,32,33]; however, reviews on phthalate and phthalate metabolite exposure are lacking. Therefore, the aim of this review was to provide an overview of published studies reporting exposure to phthalates or their metabolites and altered miRNA expression.

## 2. Methods

A literature search was conducted using PubMed, Scopus, and Web of Science to identify relevant peer-reviewed studies published in English from inception until December 2024, with additional references screened from selected articles. The search strategy combined keywords related to phthalates and their metabolites with terms related to microRNA (miRNA) and gene expression, specifically the following: (DEHP OR DBP OR BzBP OR MEHP OR MBP OR “phthalate metabolites” OR “phthalate exposure”) AND (miRNA OR microRNA OR “gene expression”). Studies were included if they investigated the effects of phthalate or phthalate metabolite exposure on miRNA expression, reported associations between altered miRNA expression and biological pathways or health outcomes, and were published in peer-reviewed journals in English. Studies were excluded if they did not specifically examine the relationship between phthalates/metabolites and miRNA, focused on other endocrine disruptors, or were review articles, editorials, conference abstracts, or letters to the editor. The research strategy yielded 77 articles; however, a full-text review found that only 44 focused on phthalates and miRNA changes. The extracted data included study design (in vitro, in vivo, epidemiological), phthalate/metabolite exposure levels, biological samples used, miRNA analysis methods (e.g., qRT-PCR, microarray), and findings on miRNA expression changes and associated health effects. Studies were grouped according to investigated health outcomes (e.g., reproductive, metabolic, neurodevelopmental effects), and results were synthesized narratively.

## 3. Phthalate/Phthalate Metabolite miRNA Dysregulation and Associated Health Effects

### 3.1. Reproductive Effects

As endocrine disruptors, phthalates and their metabolites are known to act on the hypothalamic–pituitary–gonadal (HPG) axis, impacting the normal production of sex hormones and interfering with children’s sexual development and adult sexual behaviour [34]. They influence steroid hormone release, transport, intracellular signalling, receptor activity, and gene expression [35]. Epidemiological studies link exposure to phthalates and their metabolites with reproductive health issues, including lower semen quality, altered anogenital distance, reduced testosterone and oestradiol levels, decreased antral follicle count, endometriosis, altered foetal sex hormones, and reproductive cancers [15]. Several studies have investigated the reproductive effects of phthalate exposure and their association with altered miRNA expression (Table 2).

#### 3.1.1. Placental Development and Function

Zhong, Baccarelli, Mansur, Adir, Nahum, Hauser, Bollati, Racowsky, and Machtinger [36] explored placental-derived maternal blood EV-miRNAs and phthalate exposure in uncomplicated term dichorionic, diamniotic twin pregnancies. Elevated urinary monobenzyl phthalate (MBzP) levels correlated with increased maternal blood miR-518e (ρ = 0.69, *p* = 0.03), previously linked to preeclampsia [57]. In a cohort study conducted by LaRocca, Binder, McElrath, and Michels [37], the researchers measured 11 urinary phthalate metabolites in pregnant women and analysed 29 placental miRNAs. Higher urinary phthalate levels correlated with altered miR-185 expression, with increased LMWPs significantly decreasing miR-185. In silico analysis associated miR-185 with iron ion homeostasis and small molecule metabolism signalling pathways, implicating its potential role in placental growth and preeclampsia, given the previously reported differential expression [58]. However, these changes do not appear to reflect those seen in cellular and animal work.

Gu, Liu, Luo, Wang, Wang, and Li [38] exposed pregnant Sprague Dawley (SD) rats and HTR-8/Svneo cells (100 µmol/L DEHP for 24 h) to DEHP. In vivo, DEHP caused abnormal placental histopathology and increased miR-155-5p expression. In vitro, miR-155-5p knockdown demonstrated DEHP inhibition of cell proliferation, cAMP/PKA inactivation, and enhanced lipid metabolism, suggesting miR-155-5p plays a key role in DEHP embryotoxicity. Maternal exposure effects on foetal development in this study are discussed in Section 3.4.2.

In first trimester placental HTR8/SVneo cells, MEHP induced oxidative-stress-responsive miR-17-5p, miR-155-5p, and miR-126-3p were linked to the PI3K/AKT signalling pathway [39]. These miRNAs were associated with networks involving reproductive system diseases and organismal abnormalities, suggesting a role in early pregnancy complications.

Meruvu, Zhang, Bedi, and Choudhury [40] also studied the role of miR-16 in the MEHP-induced apoptosis of HTR8/SVneo cells. MEHP increased cytotoxicity, reactive oxygen species (ROS), and miR-16 expression while decreasing cell viability and antiapoptotic BCL-2 expression. Given apoptosis is critical for placental function, the findings implicate miR-16 in apoptosis regulation via ROS.

#### 3.1.2. Ovarian/Uterine Development and Function

Zota, Geller, VanNoy, Marfori, Tabbara, Hu, Baccarelli, and Moawad [41] examined altered miRNA in fibroid tissue compared to myometrium and their associations with measured phthalates. The authors found that MHBP and MEHHP positively associated with miR-10a-5p and miR-577, respectively. From the bioinformatic analysis of 10 miRNAs, 8 were identified as having differing associations with phthalates based on race/ethnicity; miR-10a-5p, miR-128-3p (associated with MEHP), and miR-494-3p (associated with MnBP) were highlighted as having significant associations with fibroid-related processes including angiogenesis, apoptosis, differentiation of muscle cells, proliferation of connective tissues, and cell viability.

Barnett-Itzhaki, Knapp, Avraham, Racowsky, Hauser, Bollati, Baccarelli, and Machtinger [42] analysed follicular fluid phthalate metabolites and EV-miRNAs in 101 women. Among the 39 EV-miRNAs that correlated with phthalate metabolites, miR-572 was associated with 3 (MBzP, MECPP, MEP). Pathway analysis identified 20 pathways linked to ovarian development, oocyte maturation, and fertilization. Of 304 genes identified, NRG1 and ABI2 were regulated by multiple EV-miRNAs: miR143-3p, miR-193a-5p, miR-221-3p, miR-222-3p, miR25-3p, and miR-92a-3p for NRGI; miR-15a-5p, miR-25-3p, miR-376a-3p, miR-532-3p, and miR-92a-3p for ABI2. Similar associations in follicular fluid were reported by Martinez, Hauser, Liang, Mansur, Adir, Dioni, Racowsky, Bollati, Baccarelli, and Machtinger [43], suggesting miRNAs play a role in female fertility: miR-125b, miR-106b, miR-374a, and miR-15b were associated with MEHP; miR-let-7c was associated with MEHHP, MEOHP, and MECPP; the sums of metabolites of DEHP and miR-24 were associated with MBP.

In a mouse model, Liu, Wang, Zhu, Li, Luo, Huang, and Zhang [44] showed DEHP exposure reduced follicular development-related gene expression without altering protein levels or promoter methylation. Ovarian miRNAs increased nonmonotonically, with greater effect at lower levels, suppressing granulosa cell proliferation and antiapoptotic KITL/GDF9, promoting granulosa cell death via bax/bcl2. Gonsioroski, Aquino, Alonso-Costa, Barbisan, Scarano, and Flaws [45] examined maternal phthalate mixture exposure in rats; in the female offspring, they found impaired folliculogenesis and altered steroidogenesis (Star, Hsd3b1), apoptosis (Bcl2), cell cycle (Cdk4, Cdkn1a), and ovarian miR-184 expression which were found to be associated with oocyte maturation pathways.

Zhang, Zhang, Liu, Li, Shen, and Sun [46] exposed neonatal CD1 mouse ovaries to DEHP, reducing primordial follicles and increasing apoptosis, increased PTEN and Bax/Bcl2, and decreased pAKT expression. Differentially expressed miRNAs were associated with cell growth, development, and differentiation using Gene ontology (GO) analysis. MAPK, mTOR, FoxO, and oocyte meiosis and maturation pathways were found to be associated with miRNAs, suggesting a role in ovarian development.

#### 3.1.3. Prostate Development and Function

Scarano, Bedrat, Alonso-Costa, Aquino, Fantinatti, Justulin, Barbisan, Freire, Flaws, and Bernardo [47] studied perinatal phthalate mixture exposure in SD rats from gestational day 10 to postnatal day (PND) 21. Exposure (20 μg/kg, 200 μg/kg, 200 mg/kg) reduced anogenital distance, prostate weight, and testosterone at the lowest dose (PND22) and increased inflammatory foci and focal hyperplasia (PND120). Of the 120 deregulated genes, 118 were downregulated at low exposure and associated with prostate development and oncogenesis. miR-141-3p, upregulated exclusively at low exposure, and miR-184, upregulated at all treatments, were found to be associated with a significant number of the downregulated genes, suggesting that exposure impacts prostate development through the alteration of the transcriptome profile.

#### 3.1.4. Testicular Development and Function

Oluwayiose, Houle, Whitcomb, Suvorov, Rahil, Sites, Krawetz, Visconti, and Pilsner [48] examined urinary phthalate metabolites and EV-miRNAs in seminal plasma. miR-1246 was found to be positively correlated with DEHP metabolites (MEOHP, MECPP, and ƩDEHP); miR-574 was found to be positively correlated with MECPP; miR-3176 was found to be positively correlated with MHBP; and miR-7704 was found to be positively correlated with MHiNCH. Conversely, MCPP was found to be negatively correlated with miR-511. Gene targets of these miRNAs were enriched in vesicle-mediated transport and developmental pathways, including tyrosine kinase activity, head development, and cell morphogenesis. Predicted pathways involved responses to growth factor stimuli, regulation of cardiac muscle cell proliferation, and developmental processes.

Helmy, Senousy, El-Sahar, Sayed, Saad, and Elbaz [49] studied the oxidative stress effects of DEHP (500 mg/kg) on testicular tissue in Wistar albino rats, reporting decreased testis organ coefficient, 3β-HSD activity, testosterone, and Nrf2/HO-1 levels, alongside increased FABP9 levels (indicating blood–testis barrier damage), altered oxidative stress markers (MDA, GSH-Px/SOD/CAT), increased apoptosis (Bax/Bcl2), and seminiferous tubule atrophy. Targeted miRNA expression, i.e., miR-126-3p and miR-181a, were decreased and increased, respectively. miR-126-3p was found to be positively correlated with SIRT1, Nrf2, HO-1, 3-HSD, and testosterone, while miR-181a was found to be negatively correlated, suggesting that DEHP-induced testicular toxicity occurs via an oxidative stress–miR-126/miR-181a-SIRT1 network.

Tang, Zhang, Zhu, Li, Wang, Wang, Qin, and Zhang [50] observed that DBP-induced oxidative stress in SD rats increased miR-506-3p, linked to inflammation, seminiferous tubule disorganization, reduced anogenital distance, sperm count, viability, testosterone, and increased FSH/LH. Transfection studies and further analysis revealed that miR-506-3p aggravated the oxidative stress in DBP-treated rats by suppressing ANXA5 and Nrf2/HO-1 activity.

Yuan, Wu, Zhang, Gu, Guo, Jiang, and Wang [51] examined transgenerational DBP exposure effects in SD rats, finding miR-30 family downregulation and APC mRNA and protein upregulation in F3 males, associated with spermatogenesis defects.

Buñay, Larriba, Moreno, and Del Mazo [52] noted chronic phthalate exposure in C57BL/6J mice caused histopathological testis changes, reduced intratesticular oestradiol, increased Nr1h2, and downregulation of miR-18a-5p, disrupting spermatogenesis. miR-18a-5p was linked to the decrease in intratesticular oestradiol levels in the testes and was negatively correlated with Nr1h2. Yin, Ma, Han, Ding, Zhang, Han, and Li [53] investigated the association between precocious puberty and testicular tumours following low-dose MBP exposure (0.1, 1, 10 mM) in seminiferous tubule Sertoli cells (SCs) in 9-day-old male SD rats. Microarray analysis revealed nine differentially expressed miRNAs at the lowest dose. Further mRNA–miRNA network analysis identified miR-301b-3p and miR-3584-5p as critical players, with both shown to bind Rasd1 mRNA. MBP exposure increased p-MEK expression, suggesting that these miRNAs contribute to SC proliferation by downregulating Rasd1, thereby activating MEK signalling.

#### 3.1.5. Steroidogenesis and Gametogenesis

Li, Xing, Wu, Zhang, Tang, Tang, Wang, Han, Wang, Wang, Zhang, Zhou, and Qin [54] investigated DBP’s male reproductive toxicity in prenatal C57BL/6J mice (500 mg/kg/day) and in vitro using GC-1 and GC-2 germ cells (10 mg/L). In vitro, DBP exposure reduced proliferation, increased apoptosis, and caused DNA damage by downregulating the AKT pathway (decreased p-AKT, p-PI3K, mTOR, Bcl-2, and increased cleaved caspase-3, Bax) through increased PTEN expression, linked to decreased DNMT3b expression which was mediated by increased miR-29b expression. In vivo, DBP exposure altered testicular morphology, reduced sperm count, motility, and progressive sperm, and decreased sperm-flagellum-related gene expression. Bioinformatics identified miR-29b targets involved in apoptosis, proliferation, DNA methylation, and spermatogenesis. Štefánik, Michalec, Morová, Olexová, and Kršková [55] studied miR-15b-5p and miR-34a-5p expression in the hippocampus of offspring from maternal rats exposed to DEHP, DINP, and DBP (4.5 mg/kg). Results showed sex-dependent effects: miR-15b-5p was higher in females at baseline but decreased with exposure, while in males, it increased. miR-34a-5p expression decreased in pre- and perinatal exposed females. Male offspring exhibited testicular defects, highlighting sex-dependent phthalate effects into adulthood. Although the authors did not carry out target analysis, miR-15b-5p was previously found to be downregulated in mice testis that coincided with altered stereogenic gene expression [52].

Lu, Zhang, Hu, Qin, Gu, Li, Zhang, Li, and Wang [56] investigated the role of miRNA-200c in steroidogenesis in mouse Leydig tumour cells (MLTC-1) and murine adrenocortical tumour cells (Y1) exposed to MBP (10^−7^ M). MBP exposure upregulated progesterone, StAR (steroidogenic acute regulatory protein), and vimentin (involved in cholesterol transport) while downregulating miRNA-200c. Transfection analysis showed that MBP increased progesterone production via an miRNA-200c regulation of vimentin, suggesting miRNA-200c’s role in steroidogenesis.

### 3.2. Cancer

While there are conflicting findings in epidemiological studies, the consensus view is that phthalates are likely to have tumorigenic effects [59]. Recent studies have demonstrated that MECPP and DEHP metabolites are associated with increased risk of breast cancer and an increased risk of uterine leiomyoma [60]; they have also demonstrated that phthalates are associated with thyroid cancer [61] and prostate cancer [62] through androgen-receptor-dependent signalling [63]. In fact, DEHP has been shown to increase prostate-specific antigen levels while simultaneously promoting prostate cancer cells [64]. In addition, DEHP has been reported to facilitate the progression of non-small-cell lung cancer by stimulating cell proliferation and inflammation and triggering epithelial-to-mesenchymal transition [65]. BzBP exposure is reported to increase breast cancer resistance to doxorubicin/cyclophosphamide treatment and increase angiogenesis [66]. BzBP is also reported to promote breast cancer metastasis by activating the aryl hydrocarbon receptor and subsequent sphingosine kinase 1 (SPHK1)/sphingosine 1-phosphate (S1P)/sphingosine-1-phosphate receptor 3 (S1PR3) signalling [67]. Table 3 details the studies that have investigated cancer-related effects of phthalate exposure and their associations with altered miRNA expression.

#### 3.2.1. Prostate Cancer

Zhu et al. [68] studied the effects of low-dose BzBP (10−6 and 10−7 mol/L) on miR-34a expression in human prostate cancer cells (LNCaP and PC-3) for 6 days. BzBP exposure significantly decreased miR-34a expression, leading to increased cell proliferation. miR-34a downregulation coincided with increased expression of c-myc oncogene, cell cycle targeting of cyclinD1 and PCNA genes, and decreased p21 expression. The authors concluded that BzBP enhances prostate cancer cell proliferation through the miR-34a/c-myc axis.

Cavalca et al. [69] exposed LNCaP (androgen-dependent tumour cells) and PNT-2 (normal immortalized epithelial cells) to a phthalate mixture (MEP, MBP, MiBP, MBzP, MEHP, MiNP) at 1000 μmol/L. In LNCaP cells, exposure altered gene expression related to apoptosis (BAX, BCL2, CASP3, CASP8), oxidative stress (CAT, GSR, SOD1), miRNA biosynthesis and methylation markers (DNMT1, DICER1, DROSHA), and cell cycle markers (CDKN2A, CCDN2, Erα, KI67). These changes were less pronounced in normal cells, indicating enhanced migratory potential and tumour behaviour due to exposure. Additionally, miR-141-3P and miR-184 expression was increased in LNCaP cells at 24 h but not in normal cells.

#### 3.2.2. Breast Cancer

Wu et al. [70] examined the effects of BzBP treatment on miR-19a and miR-19b expression in breast cancer cells (MCF-7, ER(+) and MDA-MB-231, ER(−)). Treatment with 10^−5^ M BzBP accelerated the cell cycle from G1 to S phase, increasing proliferating cell nuclear antigen (PCNA) and Cyclin D1 levels. Transfection studies confirmed increased miR-19a and miR-19b expression in both cell lines, downregulated PTEN and p21, and upregulated p-AKT. Bioinformatics identified three binding sites of miR-19 in the PTEN 3’UT, contributing to BzBP’s promoting effects on breast cancer. The increased miR-19a and miR-19b expression due to BzBP exposure were confirmed in a similar study by Cao et al. [71], who further showed the upregulation of breast cancer stem cell markers (CD44, ALDH1A1, OCT-4) and Bcl2 levels and decreased cleaved caspase 9 expression in MCF.

#### 3.2.3. Respiratory Cancers

Wang et al. [72] studied the effects of MEHP (10nM) on human oral squamous cell carcinoma (OSCC) cells. Exposure increased cellular proliferation by upregulating PCNA and c-Myc expression and downregulating miR-27b-5p and miR-372-5p. Transfection studies confirmed that both miRNA targeted PCNA and that c-Myc downregulated miR-27b-5p. In vivo, similar results were observed in SCC-4 xenografts in male Nu/Nu mice. Reduced cellular proliferation of miR-27b by targeting FZD7 and Wnt [73] and miR-372-5p by targeting FXYD6 [74] indicate a role for these miRNA in OSCC progression.

Qin et al. [75] investigated the effects of small EV (sEV) from DEHP-exposed A549 lung epithelial cells on surrounding normal cells. At 20 μM DEHP exposure, E-cadherin was increased, mesenchymal marker N-cadherin mRNA expression was decreased, and protein levels of vimentin increased; the migration and invasion of A549 cells increased and DEHP exposure simulated the production of sEV. A total of 40 miRNAs were found to be significantly differently expressed in DEHP-exposed cells compared to controls and enriched in pathways related to cancer and tumours: TGF-β, FoxO, and HIF-1. Of the most abundant, miR-26a-5p 9 (*p* < 0.0001) and miR-125b-5p were upregulated, whereas miR-186-5p, miR-149-5p, miR-222-3p, and miR-221-3p were downregulated. Further analysis revealed that sEV derived from DEHP-treated cells induced higher miR-26a-5p expression in unexposed cells through the upregulation of the transcriptional factor twist stimulating epithelial–mesenchymal transition. The authors proposed that, by interfering with intercellular communication within cells, DEHP can cause pulmonary damage.

#### 3.2.4. Blood Cancers

Duan et al. [76] examined the effects of BzBP (10^−8^ M) on acute myeloid leukaemia (AML) cells and found increased cell proliferation, glycolysis, pyruvate dehydrogenase lipoamide kinase isozyme 4 (PDK4), and anticancer treatment desensitization. miRNA analysis revealed reduced miR-15b-5p expression, which targets PDK4, decreasing its mRNA stability. High levels of PDK are a poor prognostic marker for AML [77] and the expression of miR-15b-5p is inversely associated with PDK4 in osteosarcoma [78], HepG2 cells [79], and recurrence of hepatocellular carcinoma [80]; therefore, this study highlights an important role for the miR-15b-5p/PDK4 axis in BzBP-induced AML malignancy.

### 3.3. Metabolic Effects

Phthalates are proposed obesogens due to their potential to alter the regulation of energy balance via altering lipid homeostasis [81,82,83,84,85]. The obesogenic effects of phthalates are purported to act via several mechanisms: androgenic/anti-androgenic effects [86], impacts on the hypothalamic–pituitary–adrenal and/or –thyroid axis, and perturbed peroxisome-proliferator-activated receptors (PPAR) [82,83]. Liver enzyme levels in adolescence are impacted by childhood exposure to phthalates in daily life [87]. Phthalate exposure at human-relevant levels may exert metabolic effects, including potential implications for diabetes risk [88] and contribute to adverse cardiovascular health, with changes in BP and risk of atherosclerosis [89]. Studies that have investigated the metabolic effects of phthalate exposure and their associations with altered miRNA expression are listed in Table 4.

#### 3.3.1. Liver Function

Chen, Liu, Chen, Song, You, and Yang [90] exposed SD rats to DBP and BaP, alone or in combination, and found reduced liver miR-34a-5p expression. Exposure increased liver serum markers (ALT, AST, CHE, GLDH) and inflammatory cell infiltration, along with altered cytokine levels (IL-1β, iNOS, TGF-β, CCL22). There was also imbalance in M1/M2 macrophage polarization and upregulation of Notch signalling pathway members (Notch1, Jagged1, RBP-J, Hes1). miR-34a-5p co-localized with Notch1 in macrophages, suggesting its role in Notch signalling initiation. miR-34a-5p has been previously linked to liver damage [101].

Chorley, Carswell, Nelson, Bhat, and Wood [91] exposed male B6C3F1 mice to DEHP, DNOP, or BzBP for 7 and 28 days, finding 61 and 171 differentially expressed liver miRNAs, respectively. Of 10 significant miRNAs identified, miR-125a-5p, -182−5p, -20a−5p, and -378a−3p were linked to the PPARα pathway, with sensitivity to Acot1, Cyp4a12b, and Cyp4a14 genes. Other pathways involved Nrf2/oxidative stress (Gstm1 gene) and CAR/PXR (Abcc3 gene).

Cocci, Mosconi, and Palermo [92] exposed Gilthead seabream hepatocytes to DiDP, finding downregulation of miR-133, miR-29, and miR-199a, with increased expression of lipid-metabolism-related genes (elovl6, pla2g12b, pla2g6, cel, abhd6a). DiDP exposure increased intracellular cholesterol and triglycerides, interfering with lipid metabolism and promoting inflammation.

Liu, Chen, Chen, Ma, Cen, Wang, He, You, and Yang [93] studied the co-exposure of BaP and DBP in L02 liver cells. Exposure led to hepatocyte toxicity (elevated AST/ALT), altered proinflammatory cytokine levels (IL-2, IL-6, TNF-α, IL-10), S-phase arrest, and apoptosis. miR-122–5p was downregulated and SOCS1/STAT3 signalling was impaired. Transfection experiments revealed that miR-122–5p directly binds to 3′ -UTR of SOCS1, negatively impacting SOCS1/STAT3 signalling and resulting in observed hepatocyte injury, which was more pronounced due to the co-exposure.

#### 3.3.2. Obesity

Meruvu, Zhang, and Choudhury [94] examined miRNA-34a-5p’s role in BzBP-induced obesity in 3T3-L1 cells. BzBP doses (1, 10, and 50 μM) increased lipid content by 52%, 66%, and 90%, respectively; they also elevated PPARγ2 expression, promoting adipogenesis at a high-exposure dose. BzBP (10, 50 μM) upregulated miRNA-34a-5p, targeting the downregulation of Sirt1, Sirt3, and Nampt genes. Knockdown of miR-34a-5p reversed these effects and identified a role for NAD+ in Sirt1 activity. With miRNA-34a-5p implicated in obesity [102], the results suggest that miRNA-34a-5p expression may contribute to adipogenesis.

Zhang and Choudhury [95] investigated the effects of BzBP (50 μM) on C3H10T1/2 stem cells. BzBP exposure upregulated miR-103, miR-107, and let-7 family members while decreasing lncRNA expression of H19 target expression at day 2. Indicators of insulin signalling, i.e., insulin receptor substances (IRS1, IRS1/IRS2), were reduced, along with phospho-Akt on day 4. These findings suggest that early BzBP, through altered miRNA expression, contributes to metabolic dysregulation via insulin signalling.

#### 3.3.3. Diabetes

Wei, Hao, Chen, Li, Han, Lei, Wang, Wang, You, Chen, Li, Ding, Huang, Hu, Lin, Shen, and Lin [96] examined the role of the keap1-Nrf2 pathway and miRNAs in DEHP-induced skeletal muscle insulin resistance (SkM-I) using male C57BL/6 mice (2 mg/kg/day DEHP for 15 weeks) and C2C12 myoblasts (1, 5, and 25 µM DEHP). DEHP exposure induced insulin resistance, increased miR-200a and miR-141 expression, and decreased miR-17 expression. miR-200a upregulation was linked to oxidative stress and directly targeted Insr and Irs1, impairing Akt and GLUT4 activation and leading to insulin resistance. miR-17 downregulation, through Dnmt3a-dependent promoter methylation and lncRNA Malat1-related sponge functions, disrupted the Keap1-Nrf2 redox system, activating oxidative-stress-responsive Txnip. The study suggests that modulating miR-17 and miR-200a could have therapeutic potential for insulin resistance and type 2 diabetes.

#### 3.3.4. Cardiovascular Disease

Liu, Qin, Xu, Li, and Cong [97] exposed apolipoprotein E gene knockout C57BL/6 mice to a high-fat diet with 5% DEHP for 6 weeks. DEHP treatment increased GAS5 expression in aortic tissue, promoted atherosclerotic plaque formation, and elevated plasma triglycerides and cholesterol. Gene expression related to cholesterol metabolism was altered, with decreased LDLR, ABCA1, ABCG5, ABCG8, and SR-B1, and increased PCSK9 and ApoA2. In vitro, DEHP (100 μg/mL) exposure in vascular smooth muscle cells (VSMCs) decreased miR-145-5p expression and increased GAS5, leading to oxLDL uptake by macrophages and foam cell formation. Transfection studies showed that GAS5 competed with miR-145-5p for VSMC proliferation and apoptosis, contributing to atherosclerosis.

Wen, Kong, Cao, Xu, Zhang, Zhang, Xiao, and Wang [98], examined DBP effects on phenotypic switching in rat aortic smooth muscle (A7r5) cells. DBP (10^−7^ M) induced pebble morphology and decreased VSMC marker genes, SM-22α, α-SMA, and CNN1, confirming its role in phenotypic switching. DBP also downregulated miR-139–5p, and using miR-139–5p inhibitor, downregulation of MYOCD and VSMC marker genes was revered, suggesting DBP’s effects via the miR-139–5p-MYOCD pathway.

Kong, Wen, Cao, Xu, Zhang, Tang, Zhang, and Wang [99] investigated the effects of DBP on monocyte recruitment in EA.hy926 and THP-1 cells. DBP promoted THP-1 recruitment through an inverted U-shape dose curve with significance at 10^−8^ and 10^−7^ M. DBP upregulated MCP-1 and downregulated miR-137-3p; through further analysis using knockdown and mimic, the researchers showed that miR-137-3p binds to SP1 mRNA 3′ UTR, regulating MCP-1 and its receptor CCR2. These results highlight the role of the miR-137-3p-SP1-MCP-1 pathway in atherosclerosis development.

Wang, Wen, Xiao, Sun, Chen, Gu, Kong, Gu, Zhang, and Wang [100] examined the role of miR200c-5p in DBP-promoted lipid accumulation in THP-1 macrophages. Exposure resulted in upregulation of miR200c-5p and downregulation of mRNA levels of genes related to lipid efflux (ABCA1, ABCG1, and SR-BI), but only the protein levels of ABCA1 were reduced. Suggested binding of miR200c-5p to 3′ UTR of ABCA1 was confirmed using transfection studies. Further studies confirmed that DBP suppressed cholesterol efflux, resulting in increased lipid levels which were mediated via miR200c-5p-ABCA1 cascade, indicating a potential role in the formation of atherosclerosis.

### 3.4. Neurodevelopmental

As endocrine disruptors, the neurodevelopmental effects of phthalates are proposed as a result of altered maternal, foetal, and child testosterone and thyroid hormone levels, hormones which contribute to brain development [103]. Mechanistically, phthalates are also associated with increased oxidative stress [104], resulting in adverse birth outcomes, such as an increased risk of spontaneous preterm birth [105]. Several studies have reported adverse neurodevelopmental outcomes due to phthalate exposure. In a systematic review, prenatal maternal urinary levels of phthalate metabolites were found to be associated with adverse cognitive and behavioural development in children with sex-specific effects [106]. In addition, prenatal and childhood exposure to DEHP were found to be associated with reduced psychomotor development and intelligence quotient, respectively [107]; BzBP exposure in girls was found to be inversely associated with motor skills [108]. Similar sex-specific effects have been described in animal models exposed in utero [109]. Studies that have investigated the neurodevelopmental effects of phthalate exposure and their association with altered miRNA are listed in Table 5.

#### 3.4.1. Brain Morphology, Memory, and Learning

Luu, Green, Childers, Holahan, and Storey [110] examined hippocampal miRNA responses to acute postnatal DEHP exposure in male and female rats. In males, DEHP (1, 10, or 20 mg/kg) significantly decreased 21, 25, and 44 miRNAs, while in females, 18 miRNAs increased at 10 mg/kg exclusively. Eleven miRNAs that decreased in males increased in females, including miR-132-3p, -132-5p, -212-3p, and -212-5p, were found to be associated with dendritic regulation and spine density; miR-191a-5p was found to be linked to spine area and elimination. The results suggest that males may be more susceptible to DEHP-induced neurodevelopmental deficiencies, while females exhibit a protective effect.

Qiu, He, Zhang, Dai, Wang, Liu, Li, Hu, Xiang, and Wei [111] exposed 5-week-old male C57BL/6 mice to DEHP (0.5, 5, and 50 mg/kg/day) for 28 days to examine hippocampal pubertal effects. DEHP downregulated miR-93, leading to upregulation of TNFAIP1 which caused degradation of CK2β and inhibition of the Akt/CREB pathway, causing neuronal apoptosis (downregulated Bcl-2). Functional, molecular (BDNF, synapsin-1, PSD95), and learning and memory impairment were also observed. Through in vitro transfection studies in N2a cells, in vivo results were confirmed. Together, the results suggest DEHP-induced hippocampal pubertal effects through altered miRNA expression.

Using Zebrafish embryos exposed to 500 μg/L DEHP (2, 8, 24, and 72 h post-fertilization), Du, Yu, Huang, Zhang, Abdelhafez, Yin, Qiao, and Guo [112] revealed that downregulation of miR-146a due to exposure caused the upregulation of TRAF6 mRNA and protein, a critical factor for dendritic cell maturation and development. miR-146a was predicted to be involved in hedgehog signalling pathway, targeting HHIP, PKA, β-TrCP, and Smurf1/2 genes.

#### 3.4.2. Embryonic and Foetal Development

As discussed in Section 3.1.1 placental development, Gu, Liu, Luo, Wang, Wang, and Li [38] showed that DEHP upregulated miR-155–5p in pregnant SD rats, resulting in foetal malformations, including bulging abdomens, neck skin defects, and shorter tail lengths, suggesting miR-155–5p’s role in foetal growth restriction and malformations via cAMP/PKA inactivation. Yu, Huang, Gong, Zhang, Abdelhafez, Du, and Guo [113] studied the effects of DEHP on miR-375 and pre-mRNA processing factor 3 expression in zebrafish embryos and found that DEHP exposure (2–200 μg/L) downregulated miR-375 and upregulated PRPF3 expression, with miR-375 binding to the 3′-UTR of PRPF3 mRNA. miR-375 was linked to vascular smooth muscle contraction, TGF-β, biosynthesis of amino acids, and MAPK signalling pathways.

### 3.5. Other Effects

Although weak and inconsistent, epidemiological studies provide support for the association of phthalate exposure and allergic disease [114]. In 8-week-old female BALB/c mice, Liu et al. [115] investigated the effects of DEHP (500 mg/kg/d) exposure on the lungs of ovalbumin (OVA)-sensitized mice. There was no effect on the antibody response but DEHP exposure increased Th2 cells and decreased Th1 and Treg cells and the Th1/Th2 ratio compared to the OVA group. Inflammatory cell infiltration, thickening of airway cell layers, and shrinkage of the airway lumen were significantly increased due to DEHP exposure. miRNA analysis revealed differentially expression in the DEHP group which were enriched in the PI3K/AKT pathway, which is closely related to immune function and airway inflammation. In addition, miR-146b-5p, a possible proinflammatory factor in asthma, was upregulated with DEHP exposure and positively correlated with Th2 cells and negatively correlated with the Th1-cells and the Th1/Th2 ratio (Table 6).

## 4. Discussion

This narrative review reports on the current evidence for altered miRNA expression due to phthalate and phthalate metabolite exposure and their impacts on subsequent biological pathways. This review may provide insights into phthalate-related miRNAs as potential biomarkers of disease; in addition, their consistency, the direction of their associations, and their roles in pathophysiology are illuminated.

Considerable variability was noted in the reviewed studies, encompassing differences in the examined phthalates and their doses, the types of tissue samples used, the methodologies for miRNA analysis, and the specific study designs. There were a limited number of studies in humans (six studies), of which most focussed on female reproductive health. In vitro studies in human cell lines (14 studies) focussed on placental function (HTR-8/Svneo cells) and breast (MCF-7), prostate (LNCaP), and other cancers, as well as cardiovascular disease and liver function. The most common in vivo model involved SD rats (eight studies), with most animal studies focusing on male reproductive effects. The methodology that was most often used for miRNA analysis was qRT-PCR, utilized in thirty-nine studies, likely for its precision, accuracy, speed, and accessibility [116]. DEHP emerged as the most extensively investigated phthalate, garnering a total of nineteen studies, while DBP and BzBP exposure were examined in ten and eight studies, respectively. miRNA expression due to phthalate metabolite exposure was examined in eleven studies in total, with MEHP and MBP being the most examined (accounted for in seven and four studies, respectively). The prominence of DEHP and DBP as the most extensively studied phthalates can be attributed to their widespread usage in plastic manufacturing, their common detection in biomonitoring studies [4], and their associated detrimental health effects [7], leading to enhanced regulations [13]. 

The exposure concentrations ranged from very low concentrations in the nanomolar to micromolar range. Notably, low-dose exposures, such as DBP (10^−9^–10^−5^ mol/L) and BzBP (10^−7^–10^−3^ M), are within the range of human exposure levels reported in biomonitoring studies [71,99], making them relevant to real-world scenarios. Of the studies reviewed, 20 studies used low concentrations (nanomolar to micromolar range) and reported altered miRNA expression. These low concentrations were shown to induce significant miRNA dysregulation, suggesting that even environmentally relevant levels of phthalate exposure can disrupt miRNA expression and contribute to adverse health outcomes. High-dose exposures, such as DEHP at 1600 mg/kg/day, were also used, although these concentrations typically exceed human exposure levels [44]. Furthermore, some studies reported nonmonotonic dose–response curves, where greater effects on miRNA expression were observed at lower concentrations [44,47]. This pattern is consistent with the complex dose–response relationships often observed with endocrine-disrupting chemicals, where low doses can sometimes elicit stronger biological effects than higher doses, underscoring the need for further research into the mechanisms and health risks associated with chronic, low-level phthalate exposure.

In general, miRNAs were upregulated more often than downregulated on exposure to phthalates and their metabolites (Table 2, Table 3, Table 4, Table 5 and Table 6). Given that miRNAs typically function as inhibitors of gene expression by binding to and suppressing their target mRNAs [24], the upregulation of miRNAs would likely enhance their inhibitory effects. This could lead to a reduction in the expression of critical genes, potentially disrupting cellular functions and contributing to the adverse health effects associated with phthalate exposure. miR-34a, miR-15b, miR-141 were the most common miRNAs, as they were observed in four different studies. However, their regulation was not always consistent. miR-34a was downregulated in prostate cancer and liver toxicity but upregulated in adipogenesis, indicating context-dependent roles [68,90,94,96]. Similarly, miR-15b was downregulated in AML and testicular toxicity but showed sex-dependent changes in neurodevelopmental studies [52,55,76]. In contrast, miR-141 was consistently upregulated in prostate-related studies, suggesting a stable role in prostate development and cancer progression [47,69]. These findings highlight the complexity of miRNA regulation, which can vary based on tissue type, sex, and specific phthalate exposure conditions. miR-34a is highly expressed in the adult mammalian brain [117]; it is a tumour suppressor [118], a regulator of endothelial and mitochondrial function [119], a modulator of NF-κB signalling pathway for T cell function [120], and appears to function as a negative regulator of SERPINA3 in preeclampsia [121]. miR-15 plays a pivotal role in regulating T cell responses and maintaining immune homeostasis; studies have shown that the absence of miR-15/16 leads to an increased generation of memory T cells [122]. Additionally, overexpression of miR-15b induces cell cycle arrest in the G0/G1 phase, highlighting its significant role in regulating cell cycle progression, particularly in glioma cells [123], thereby acting as a tumour suppressor [124]. In contrast, miR-15b has been implicated in promoting breast cancer progression by targeting and inhibiting the tumour suppressor gene PAQR3 [125]. Moreover, miR-15b-5p plays a detrimental role in sepsis by inhibiting SIRT4, thereby contributing to inflammation, oxidative stress, and endothelial dysfunction [126]. miR-141-3p demonstrates dual roles in cancer progression, acting as both a tumour suppressor and an oncogene. As a tumour suppressor, miR-141-3p inhibits cell proliferation, migration, and invasion in non-small-cell lung cancer [127], in chronic myeloid leukaemia by targeting RAB32 [128], and in triple-negative breast cancer by suppressing the Wnt/β–catenin signalling pathway [129]. Additionally, miR-141-3p suppresses glioma growth by targeting and inhibiting the antiapoptotic factor ATF5 [130]. Conversely, miR-141-3p contributes to retinoblastoma progression [131] and promotes autophagy in trophoblast cells under hypoxic conditions [132]. In gallbladder cancer, plasma miR-141 levels serve as a valuable biomarker for diagnosis and prognosis, effectively distinguishing patients from healthy controls while also promoting oncogenic activity [133]. Similarly, miR-141-3p acts as an oncogene in colon cancer by targeting and inhibiting Sirt1, a tumour suppressor [134]. Additionally, miR-184, miR-19a, miR-125, and miR-let-7 were also observed to be recurrent in three separate studies each. These findings underscore the potential importance of these miRNAs in regulatory pathways and suggest that they may play crucial roles in various biological processes that are worthy of further investigation.

The PI3K, Bax/Bcl2, TGF-β, Wnt, TNF-α, C-MYC, PTEN, MAPK/ERK1/2, and Nrf2 signalling pathways were commonly involved in various studies and have significant implications for different biological processes and diseases. The PI3K/AKT pathway is critical for orchestrating the stages of red blood cell development [135], serves as a key regulator of the survival, growth, and metastatic potential of cancer stem cells [136], and protects cardiomyocytes from apoptosis [137]. Additionally, targeting this pathway holds promise as a potential therapeutic strategy for preeclampsia [138]. The Bax/Bcl2 ratio mediates apoptosis by regulating mitochondrial integrity, and its dysregulation is implicated in both cancer and neurodegenerative diseases [139]. TGF-β is a multifunctional cytokine and a key therapeutic target in fibrosis and cancer [140]. Wnt signalling is a critical regulator of tumorigenesis and the tumour microenvironment; targeting this pathway enhances the efficacy of cancer immunotherapy [141]. Its dysregulation is also linked to the development of osteoporosis, as its essential for maintaining bone mass and density [142]. TNF-α, a key cytokine, plays a significant role in autoimmune diseases, with TNF-α inhibitors revolutionizing their treatment [143]; they achieve this by impairing insulin signalling by increasing the serine phosphorylation of insulin receptor substrates, leading to decreased insulin sensitivity [144]. C-MYC is a proto-oncogene that can lead to unchecked cellular proliferation, a hallmark of many cancers [145]. PTEN negatively regulates the PI3K/Akt pathway by dephosphorylating PIP3, with its dysregulation linked to metabolic disorders like diabetes and obesity [146]. ERK1/2, a key member of the MAPK family, promotes cell survival when activated by growth factors or oestrogen; however, its activation by cytokines or free radicals can also drive neurotoxic effects, such as inflammation and oxidative stress in ischemic damage [147]. Nrf2 is essential for combating oxidative stress, offering potential as a therapeutic target to prevent doxorubicin-induced cardiotoxicity [148]. However, it may also promote resistance to chemotherapy and cancer treatments [149]. Activation of the Nrf2 pathway shows promise in mitigating cerebral ischemia/reperfusion injury, protecting the blood–brain barrier, and supporting mitochondrial homeostasis [150].

Phthalates have been linked to several adverse reproductive effects, including increased risk of uterine leiomyoma in females and changes in testicular tumour development in males. These effects are mediated, in part, by alterations in miRNA expression, which have been shown to impair spermatogenesis and reduce fertility [41,49,50,51,53,54]. Studies on placental-derived EV-miRNAs suggest that miRNAs may serve as novel endocrine pathways that are sensitive to phthalate exposure [36]. Beyond reproductive effects, phthalate exposure has been associated with obesity [94], and miRNAs such as miR-34a and miR-15b-5p have been identified as potential regulators of adipogenesis and lipid metabolism [76,90,94]. Additionally, phthalates influence miRNA expression in cancer cells, suggesting a potential link to cancer development. For example, BzBP exposure alters miRNA expression in prostate cancer cells, breast cancer cells, and acute myeloid leukaemia (AML), promoting cell proliferation and tumour progression [68,70,76,94]. DBP and BzBP appear to play a significant role in the development of atherosclerosis affecting the processes involved in monocyte recruitment and vascular smooth muscle cell phenotypic switching [94,98,99].

This review highlights that altered miRNA expression due to phthalate exposure is implicated in a wide range of health conditions, including reproductive effects, cancer, metabolic disorders, and neurodevelopmental outcomes. Although research on the effects of phthalates on miRNA expression is still in its infancy, these chemicals appear to have a significant impact on miRNA expression. This review is subject to limitations, including the heterogeneity of study designs, exposure levels, and miRNA analysis methods, which have limited direct comparisons and precluded meta-analysis. The review relied on published data, which may introduce publication bias, and restricting the search to English-language studies may have excluded relevant research. In addition, there were limited number of human studies, resulting in the predominance of animal models, with most human studies relying on circulating miRNA in plasma or fluid which may not always accurately represent the miRNA profiles of target tissues [151]. Though miRNA expression in human versus rodent pluripotent stem cells reveals significant similarities, species-specific miRNAs contribute to unique regulatory aspects of human pluripotency [152], potentially limiting direct extrapolation to humans. Many of the results are extrapolated either by the presence of detected phthalates (and therefore potentially an epiphenomenon) or a cellular response in vitro to a specific phthalate that may or may not occur in vivo; however, this review brings the literature together, the weight of which suggests that phthalates have epigenetic effects that deserve further investigation. Finally, not all in vitro studies used knockdown to verify the relationship between miRNA and target genes; therefore, possible relationships are suggestive rather than conclusive.

## 5. Conclusions

This study highlights that several miRNAs are dysregulated following exposure to phthalates and their metabolites. Notably, while some miRNAs, such as miR-141, exhibit consistent expression changes across studies (e.g., upregulation in prostate-related conditions), others, like miR-34a and miR-15b, show context-dependent regulation, varying by tissue type, phthalate exposure levels, and biological context. More research involving human participants is essential for validating findings from animal models and assessing the real-world implications of phthalate exposure, at environmental exposure levels, on miRNA dysregulation. In vitro research combined with human studies would support better understanding of the mechanisms of action of miRNA in pathological processes. Additionally, integrating mRNA target analysis with miRNA analysis would be beneficial in understanding the downstream effects of phthalate exposure on gene expression and grasping the broader biological implications. Validating the effects of phthalates on miRNAs linked to reproduction and cancer is necessary given their potential adverse health outcomes.

## Figures and Tables

**Table 1:** Common phthalates and their metabolites.

Parent Phthalate	Metabolites	TDI [13] (mg/kg/Day)
High molecular weight		
DEHP (Di(2-ethylhexyl) phthalate)	Mono(2-ethylhexyl) Phthalate (MEHP) Mono(2-ethyl-5-hydroxyhexyl) phthalate (MEHHP) Mono(2-ethyl-5-oxohexyl) phthalate (MEOHP) Mono(2-ethyl-5-carboxypentyl) phthalate (MECPP)	0.05
DnOP (Di-n-octyl phthalate)	Mono-n-octyl phthalate (MnOP)	NA
DiNP (Di-isononyl phthalate)	Mono-isononyl phthalate (MiNP)	0.15
DiDP (Di-isodecyl phthalate)	Mono-isodecyl phthalate (MiDP)	0.15
Low molecular weight		
DMP (Di-methyl phthalate)	Mono-methyl phthalate (MMP)	0.05
DEP	Mono-ethyl phthalate (MEP)	
BzBP (Benzylbutyl phthalate)	Mono-benzyl phthalate (MBzP)	0.5
DBP (Di-n-butyl phthalate)	Mono-n-butyl phthalate (MnBP) Mono-3-hydroxybutyl phthalate (MHBP) Mono-(3-carboxypropyl) phthalate (MCPP)	0.01
DiBP (Di-isobutyl phthalate)	Mono-isobutyl phthalate (MiBP)	NA

TDI, tolerable daily intake; NA, no available.

**Table 2:** Phthalate-associated miRNA and reproductive effects.

Phthalate ^a^ Level/^b^ Dose	Study Design; ^c^ Sample (n)	miRNA	miRNA Expression	Methodology	miRNA Target Analysis	Signalling Pathways; Target Genes	Ref.
MBzP (2.5 ng/mL)	Pilot study; maternal blood plasma (n = 10)	miR-518e	↑	Microarray	Bioinformatic analysis, DIANA-miRPath	TGF-β, proteoglycans in cancer and transcriptional misregulation in cancer	[36]
DEHP, DOP, DiNP, DiDP, BzBP, DiBP, DnBP, DEP (dose N/S)	Cohort study; placenta samples (n = 48)	miR-185	↓	qRT-PCR	Bioinformatic analysis, miRWALK, miRanda, miRDB, TargetScan, RNA22, Gene ontology (GO), genome-wide expression	Protein serine/threonine kinase, apoptotic signalling, insulin signalling, regulation of metencephalon development, and embryonic epithelial tube formation, iron ion homeostasis and small molecule metabolic process	[37]
DEHP (9 days × 100, 500, and 1000 mg/kg/day) DEHP (24 h × 25, 50, or 100 µmol/L)	In vivo; Sprague Dawley (SD) rats; placentae (n = 8), in vitro; Trophoblasts HTR-8/Svneo cells (N/S)	miR-155–5p	↑	qRT-PCR	Western blot, ELISA, qRT-PCR	cAMP/PKA signalling; SREBP1, PPARG, FASN, ACC, ACLY, SCD1	[38]
MEHP (48 h × 25, 50, 100, and 180 μM)	In vitro; HTR-8/SVneo cell line (n = 3)	miR-17-5p	↑	qRT-PCR	qRT-PCR, bioinformatic analysis, Ingenuity Pathway Analysis (IPA)	I3K/AKT, PTEN, and NRF2-mediated oxidative stress response signalling; PIK3R1, PTEN, CDKN2A, DHCR24, SOD2	[39]
		miR-126-3p	↑				
		miR-155-5p	↑				
MEHP (48 h × 180 μM)	In vitro; HTR-8/SVneo cell line (n = 3)	miR-16	↑	qRT-PCR	qRT-PCR, Western blot	BCL-2, BAX	[40]
MHBP, MEHHP, MEHP, MnBP (1.06, 6.39, 1.7, 8.65 ng/mL)	Cross-sectional study; fibroid tumour (n =19)	miR-10a-5p	↓	TaqMan OpenArray, qRT-PCR	Bioinformatic analysis, IPA, TarBase, Target Scan, miRecords	Fibroid-related processes; BMI1, E2F3, HMO1, KIT, KLF4, NF1, NTRK3, PTEN, SNAP25, TGFBR1, TXNIP, WEE1, ADORA2B, DBI, FGF16, KMT2A, AGO1, HOA1 LDLR, VSNL1, WEE1, HOD10, USF2, AFF1, DC, RELN, SCN3A	[41]
		miR-577	↑				
		miR-128-3p	ND				
		miR-494-3p	ND				
MEP, MnBP, MiBP, MBzP MCPP, MEHP, MEHHP MEOHP, mECPP, MiNP MCOMHP, MCOMOP (median 0–1.7 ng/mL)	Prospective study; follicular fluid EV-miRNA (n = 101)	39 EV-miRNA	N/S	Microarray	Bioinformatic analysis, miRWalk, DAVID, and Kyoto Encyclopaedia of Genes and Genomes (KEGG)	Ovary, oocyte development, oocyte maturation, and fertilization; NRG1, ABI2	[42]
MEHP, MEHHP, MEOHP MECPP, DEHP, MBP (3.69, 13.49, 9.71, 20.16, 0.16, 19.01 μg/L)	Cross-sectional, nested case–control design; follicular fluid EV-miRNA (n = 130)	miR-125b	↓	Microarray	Bioinformatics analysis, DIANAmT, miRanda, miRDB, miRWalk, PICTAR5, RNA22, Targetscan	Follicular development and oocyte maturation and function: TGF-beta, endocrine resistance, PI3K-Akt, focal adhesion, FoxO, cell cycle, MAPK, EGFR tyrosine kinase inhibitor resistance, prolactin, p53, HIF-1, ErbB, cAMP, oocyte meiosis, progesterone-mediated oocyte maturation, ubiquitin mediated proteolysis, and Jak-STAT; mapk1, akt3, map2k1, pik3r3, and raf1	[43]
		miR-106b	↓				
		miR-374a	↓				
		miR-15b	↓				
		miR-let-7c	↓				
		miR-24	↓				
DEHP (6 weeks × 100, 400, 1600 mg/kg/d)	ICR mice; ovarian tissue (n = 6)	let-7b	↑	qRT-PCR	qRT-PCR, Western blot	kitl, c-kit, Gdf9, Atm, Bax, Bcl2	[44]
		miR-17-5p	↑				
		miR-181a	↑				
		miR-151	↑				
DEP, DEHP, DBP, DiNP, DiBP, and BzBP (20 μg/kg/day, 200 μg/kg/day, 200 mg/kg/day)	Pregnant SD rats; ovarian tissue (n = 4–8)	miR-184	↑	qRT-PCR	qRT-PCR, ELISA, bioinformatic analysis, miRWalk, KOBAs, KEGG, GO	Oocyte meiosis and progesterone-mediated maturation pathways; Bcl2, Cdk4, Cdkn1a, Star, Hsd3b1	[45]
DEHP (72 h × 10, and 100 μM)	In vitro; CD1 mice ovary (n = N/S/3)	miR-19a-3p	↓	miRNA Sequencing, qRT-PCR	Western blot, TUNEL Staining, miRWalk, GO	PI3K/AKT1/mTOR signalling pathways; PTEN, Bax/Bcl2, pAKT	[46]
		miR-141-3p	↓				
		miR-32-5p	↓				
DEHP (DG10—DPN21 × 20, 200 μg/kg/day, 200 mg/kg/day)	SD rats; prostate tissue (n = 3)	miR-30d-5p	↑	miRNA sequencing	mRNA sequencing, bioinformatic analysis, miRWalk, GO	Prostate development and oncogenesis; Cited1, Crmp1, Fabp5, Fez1, Hoxd3, Lcn2, Ngfr, Pcp4, Ptgr1, Qsox1, Rtn1, Tmeff2, Trpc6, Tubb3, Wnt9b, Wt1	[47]
		miR-30b-5p	↑				
		miR-141-3p	↑				
		miR-30d-3p	↑				
		miR-184	↑				
MEOHP, MECPP, MHBP, MHiNCH, ƩDEHP (3.94, 8.84, 0.72, 1.14, 0.07 μg/L)	Cohort study; seminal plasma (n = 96)	miR-1246	↑	miRNA sequencing	TargetScan, miRTarBase, PITA-MINER, miRWalk, multiMiR, tRFTars, BEDTools, metascape, STRING	MAPK1, BMPR1A/2, PTEN, TGFBR2, TP53, and APP	[48]
		miR-574	↑				
		miR-3176	↑				
		miR-7704	↑				
		miR-511	↓				
DEHP (30 days × 500 mg/kg)	Male Wistar albino rats; testicular tissue (n = 8)	miR-126-3p	↓	qRT-PCR	qRT-PCR, ELISA, Western blot	SIRT1, Nrf2, HO-1, 3-HSD, GSH-Px, SOD, CAT, 3β-HSD, FABP9, MDA, Bax/Bcl2	[49]
		miR-181a	↑				
DBP (2 weeks × 800 mg/kg/day) (10 mg/L)	SD rats; testicular cells (n = 3), in vitro; mouse Leydig TM3 cells (n = 3)	miR-506-3p	↑	Microarray, qRT-PCR	Microarray Assay, Dual-Luciferase Reporter Assay, bioinformatic analysis, Western blot, immunohistochemistry	ANXA5, Nrf2, HO-1, NQO1, GST, MDA, CAT, T-AOC, SOD, T-GSH, reduced GSH	[50]
DBP (7 days × 500 mg/kg)	F3 males of maternal exposed SD rats; testicular tissue (n = 3)	miR-30	↓	Microarray	RNA sequencing, Western blot, bioinformatics analysis, GO, KEGG	Wnt, colorectal cancer, endometrial cancer, regulation of actin cytoskeleton, cellular morphogenesis, cytoskeleton organization, basal cell carcinoma, cytoskeletal protein binding, and biogenesis signalling pathway; APC gene	[51]
DEHP, DBP, BzBP (60 days × 0.3 mg/kgday)	C57BL/6J mice; testicular tissue (n = 3)	miR-7686-5p	↑	miRNA sequencing, qRT-PCR	qRT-PCR, immunohistochemistry, Western blot, bioinformatic analysis, miRWalk database, DIANA-TarBas, IPA, GO	Hormonal signalling, genitalia development, cell proliferation, programmed cell death, histone H3-K4 trimethylation, protein folding, RNA polymerase transcription factor activity, and phosphatidylinositol phosphatase activity; Nr1h2, Star, Cyp17a1, Sp1, Cyp11a1, and Cyp19a1	[52]
		miR-34b-5p	↑				
		miR-18a-5p	↓				
		miR-15b-5p	↓				
		miR-1981-5p	↓				
		miR-382-5p	↓				
		miR-20b-5p	↓				
		miR-1291	↓				
		miR-378b	↓				
		miR-3085-3p	↓				
MBP (24 h × 0.1, 1, 10 mM)	SD rats; Sertoli cells (n = 6)	miR-301b-3p	↑	Microarray, qRT-PCR	Microarray, qRT-PCR, Western blot, Luciferase reporter assay, bioinformatic analysis, GO, KEGG	MEK signalling; p-MEK, Rasd1	[53]
		miR-3584-5p	↑				
DBP (500 mg/kg/day) (24 h × 10 mg/L)	Male 8-week-old C57BL/6J mice; testicular tissue (n = 6), in vitro; GC-1 and GC-2 cells (N/S)	miR-29b	↑	miRNA sequencing	qRT-PCR, Western blot, Luciferase reporter assay	p-AKT, p-PI3K, mTOR, and Bcl-2, cleaved caspase-3, Bax, Bax/Bcl-2, γ-H2AX, Cfap43, Cfap44, Dnah1, Ddx4, Mne8, Akap4, Ccdc39, Eno4, Fsip2, DNMT3b, Ddx39a, Wnt16, and Ebf	[54]
DEHP, DINP, and DBP (29 days × 4.5 mg/kg/day)	Wistar rats; hippocampal tissue (n = 6)	miR-15b-5p	↑♂ ↓♀	FISH	N/A	N/P	[55]
		miR-34a-5p	↓♂ ↑♀				
MBP (24 or 48 h × 10^−7^ M)	In vitro; MLTC-1 and Y1 cells (n = 3)	miR-200c	↓	qRT-PCR	qRT-PCR, Western blot	StAR, vimentin	[56]

Note: ^a^ refers to mean level detected in human studies; ^b^ refers to dose used in in vivo/in vitro studies; ^c^ refers to miRNA detection; not performed (N/P); not specified (N/S); ND, no difference; ♂, male; ♀, female; ↑, upregulation; ↓, downregulation.

**Table 3:** Phthalate-associated miRNA and cancer effects.

Phthalate ^a^ Level/^b^ Dose	Study Design; ^c^ Sample (n)	miRNA	miRNA Expression	Methodology	miRNA Target Analysis	Signalling Pathways; Target Genes	Ref.
DBP (2 weeks × 800 mg/kg/day) (10 mg/L)	SD rats; testicular cells (n = 3), in vitro; mouse Leydig TM3 cells (n = 3)	miR-506-3p	↑	Microarray, qRT-PCR	Microarray Assay, Dual-Luciferase Reporter Assay, bioinformatic analysis, Western blot, immunohistochemistry	ANXA5, Nrf2, HO-1, NQO1, GST, MDA, CAT, T-AOC, SOD, T-GSH, reduced GSH	[50]
DBP (7 days × 500 mg/kg)	F3 males of maternal exposed SD rats; testicular tissue (n = 3)	miR-30	↓	Microarray	RNA sequencing, Western blot, bioinformatics analysis, GO, KEGG	Wnt, colorectal cancer, endometrial cancer, regulation of actin cytoskeleton, cellular morphogenesis, cytoskeleton organization, basal cell carcinoma, cytoskeletal protein binding and biogenesis signalling pathway; APC gene	[51]
DEHP, DBP, BzBP (60 days × 0.3 mg/kgday)	C57BL/6J mice; testicular tissue (n = 3)	miR-7686-5p	↑	miRNA sequencing, qRT-PCR	qRT-PCR, immunohistochemistry, Western blot, bioinformatic analysis, miRWalk database, DIANA-TarBas, IPA, GO	Hormonal signalling, genitalia development, cell proliferation, programmed cell death, histone H3-K4 trimethylation, protein folding, RNA polymerase transcription factor activity, and phosphatidylinositol phosphatase activity; Nr1h2, Star, Cyp17a1, Sp1, Cyp11a1, and Cyp19a1	[52]
		miR-34b-5p	↑				
		miR-18a-5p	↓				
		miR-15b-5p	↓				
		miR-1981-5p	↓				
		miR-382-5p	↓				
		miR-20b-5p	↓				
		miR-1291	↓				
		miR-378b	↓				
		miR-3085-3p	↓				
MBP (24 h × 0.1, 1, 10 mM)	SD rats; Sertoli cells (n = 6)	miR-301b-3p	↑	Microarray, qRT-PCR	Microarray, qRT-PCR, Western blot, Luciferase reporter assay, bioinformatic analysis, GO, KEGG	MEK signalling; p-MEK, Rasd1	[53]
		miR-3584-5p	↑				
DBP (500 mg/kg/day) (24 h × 10 mg/L)	Male 8-week-old C57BL/6J mice; testicular tissue (n = 6), in vitro; GC-1 and GC-2 cells (N/S)	miR-29b	↑	miRNA sequencing	qRT-PCR, Western blot, Luciferase reporter assay	p-AKT, p-PI3K, mTOR and Bcl-2, cleaved caspase-3, Bax, Bax/Bcl-2, γ-H2AX, Cfap43, Cfap44, Dnah1, Ddx4, Mne8, Akap4, Ccdc39, Eno4, Fsip2, DNMT3b, Ddx39a, Wnt16, and Ebf	[54]
DEHP, DINP, and DBP (29 days × 4.5 mg/kg/day)	Wistar rats; hippocampal tissue (n = 6)	miR-15b-5p	↑♂ ↓♀	FISH	N/A	N/P	[55]
		miR-34a-5p	↓♂ ↑♀				
MBP (24 or 48 h × 10^−7^ M)	In vitro; MLTC-1 and Y1 cells (n = 3)	miR-200c	↓	qRT-PCR	qRT-PCR, Western Blot	StAR, vimentin	[56]

Note: ^a^ refers to mean level detected in human studies; ^b^ refers to dose used in in vivo/in vitro studies; ^c^ refers to miRNA detection; not performed (N/P); not specified (N/S); ♂, male; ♀, female; ↑, upregulation; ↓, downregulation.

**Table 4:** Phthalate-associated miRNA and metabolic effects.

Phthalate ^a^ Level/^b^ Dose	Study Design; ^c^ Sample (n)	miRNA	miRNA Expression	Methodology	miRNA Target Analysis	Signalling Pathways; Target Genes	Ref.
DBP (90 days × 50, 250 mg/kg)	SD rats; liver tissue (n = 6)	miR-34a-5p	↓	qRT-PCR	qRT-PCR, Western blot, Luciferase reporter assay, ELISA, FISH	Notch1; ALT, AST, CHE, GLDH, IL-1β, iNOS, TGF-β, CCL22, Arg, Notch1, Jagged1, RBP-J and Hes1	[90]
DEHP (7 days × 750, 1500, 3000, and 6000 ppm), DNOP (7 days × 1250, 2500, 5000, and 10,000 ppm), or BzBP (7 days × 1500, 3000, 6000, and 12,000 ppm)	B6C3F1 mice; serum and liver tissue (n = 6–8)	miR-182−5p miR-378a−3p miR-125a−5p miR-194−2-3p miR-20a-5p miR-320−3p miR-339−5p miR-423−5p miR-455−3p miR-98−5p	↑ ↑ ↑↓ N/S	miRNA sequencing, ddPCR (serum)	Bioinformatic analysis, IPA, TargetScan, TarBase	PPARα, Nrf2, CAR/PXR pathway; Acot1, Cyp4a12b, Cyp4a14, Gstm1, Abcc3	[91]
DiDP (48 h × 0.1, 1.0 and 10 μM)	In vitro; Sparus aurata hepatocytes (n = 3)	miR-133	↓	qRT-PCR	qRT-PCR, Bioinformatic analysis, TargetScan Fish	Hepatic fatty acid synthesis, glycerolipid/glycerophospholipid metabolism, phospholipid remodelling signalling pathways; cel, elovl6, pla2g6, pla2g12b, abhd6a, and pxr	[92]
		miR-199a	↓				
		miR-29	↓				
BaP (12.5 mmol/L) and/or DBP (25 mmol/L)	In vitro; human normal liver cell line (L02 cell line) (n = 3)	miR-122–5p	↓	qRT-PCR	qRT- PCR, Western blot, ELISA, Luciferase reporter assay	SOCS1/STAT3 signalling; AST, ALT, IL-2, IL-6, TNF-α, IL-10, SOCS1, STAT3	[93]
BzBP (50 μM)	In vitro; 3T3-L1 cells (n = 3)	miR-34a-5p	↑	qRT-PCR	qRT-PCR, Western blot	PPARγ2, Sirt, Sirt3, Nampt, NAD+	[94]
BzBP (8 days × 0.1 μM, 50 μM)	In vitro; C3H10T1/2 cell line (n = 3)	miR-103	↑	qRT-PCR	Western blot, Bioinformatic analysis, IPA, TargetScan	Phospho-Akt and insulin signalling pathway; H19, IRS-1, IRS-2, Akt, aP2, FoxO1, PPARγ	[95]
		miR-107	↑				
		miR-let-7(a-d, g-f)	↑				
DEHP (15 weeks × 2 mg/kg/day) ((0, 1, 5, and 25 µM))	Male C57BL/6 mice; skeletal muscle (n = 4), in vitro; C2C12 myoblast cells (n = 4)	miR-200a	↑	qRT-PCR	qRT-PCR, Western blot, Luciferase reporter assay	Insulin signalling; Insr, Irs1, Akt, Keap1, Nrf2, Dnmt3a, Malat1, Txnip, Glut4	[96]
		miR-141	↑				
		miR-17	↓				
DEHP (6 weeks × 5%w/w) (100 μg/mL)	C57BL/6 mice; aortic tissue (n = 3), in vitro; VSMCs (n = 3)	miR-145-5p	↓	qRT-PCR	qRT-PCR, Western blot, Luciferase reporter assay	LDLR, ABCA1, ABCG5, ABCG8, SR-B1, PCSK9, ApoA2, GAS5	[97]
DBP (24 h × 10^−7^ M)	In vitro; rat aortic smooth muscle (A7r5) cells (n = 3)	miR-139–5p	↑	qRT-PCR	qRT-PCR, Western blot, Luciferase reporter assay	SM-22α, α-SMA, CNN1, MYOCD	[98]
DBP (24 h × 10^− 9^–10^− 5^ mol/L)	In vitro; EA.hy926 (n = N/S)	miR-137-3p	↓	qRT-PCR	qRT-PCR, Western blot, ELISA, Luciferase reporter assay	SP1, MCP-1	[99]
DBP (24 h × 10^−7^ mol/L)	In vitro; THP-1 macrophages (n = N/S)	miR-200c-5p	↑	qRT-PCR	qRT-PCR, Western blot, Luciferase reporter assay, Bioinformatic analysis, Targetscan, RNAhybrid	ABCA1, ABCG1, and SR-BI	[100]

Note: ^a^ refers to mean level detected in human studies; ^b^ refers to dose used in in vivo/in vitro studies^; c^ refers to miRNA detection; not specified (N/S); ↑, upregulation; ↓, downregulation.

**Table 5:** Phthalate-associated miRNA and neurodevelopmental effects.

Phthalate ^a^ Level/^b^ Dose	Study Design; ^c^ Sample (n)	miRNA	miRNA Expression	Methodology	miRNA Target Analysis	Signalling Pathways; Target Genes	Ref.
DEHP (males (20 mg/kg), females (10 mg/kg))	Long Evans rat; hippocampal tissue (n = 4)	miR-132-3p	↓♂ ↑♀	qRT-PCR	N/P	Brain-derived neurotrophic factor (BDNF), cAMP response element binding protein (CREB) signalling pathway	[110]
		miR-132-5p	↓♂ ↑♀				
		miR-212-3p	↓♂ ↑(♀				
		miR-212-5p	↓♂				
		miR-191a-5p	↓♂ ↑♀				
DEHP (28 days × 0.5, 5, and 50 mg/kg/day) (100 μM)	C57BL/6 mice; hippocampal tissue (n = 9), in vitro; N2a cells (n = 9)	miR-93	↓	qRT-PCR	ELISA, immunohistochemistry, Luciferase reporter assay, Western blot	TNFAIP1, CK2β, Akt, CREB, Bcl-2, BDNF, synapsin-1, PSD95	[111]
DEHP (8, 24, and 72 hpf × 500 μg/L)	In vitro; Zebrafish; embryos (n = 3)	MiR-146a	↓	miRNA sequencing, qRT-PCR	Luciferase reporter assay, Western blot, bioinformatic analysis, TargetScanFish, DIANA-microT-CDS, miRanda, GO, KEGG	Hedgehog pathway; HHIP, PKA, β-TrCP, Smurf1/2, TRAF6	[112]
DEHP (9 days × 100, 500, and 1000 mg/kg/day) DEHP (24 h × 25, 50, or 100 µmol/L)	In vivo; SD rats; placentae (n = 8), in vitro; Trophoblasts HTR-8/Svneo cells (N/S)	miR-155–5p	↑	qRT-PCR	Western blot, ELISA, qRT-PCR	cAMP/PKA signalling; SREBP1, PPARG, FASN, ACC, ACLY, SCD1	[38]
DEHP (24 h × 2, 10, 50, 100, and 200 μg/L)	In vitro; Zebrafish embryos (ZF4) cells (n = 3)	miR-375	↓	qRT-PCR	qRT-PCR, Western blot, Luciferase reporter assay, bioinformatic analysis, DAVID, Metascape, WebGestalt, GO, KEGG, DIANA-mi-croT-CDS, miRanda, miRwalk	Vascular smooth muscle contraction, TGF-β, biosynthesis of valine, leucine, and isoleucine, MAPK signalling pathway; PRPF3	[113]

Note: ^a^ refers to mean level detected in human studies; ^b^ refers to dose used in in vivo/in vitro studies; ^c^ refers to miRNA detection; not performed (N/P); not specified (N/S); ♂, male; ♀, female; ↑, upregulation; ↓, downregulation.

**Table 6:** Phthalate-associated miRNA and other health effects.

Phthalate ^a^ Level/^b^ Dose	Study Design; ^c^ Sample (n)	miRNA	miRNA Expression	Methodology	miRNA Target Analysis	Signalling Pathways; Target Genes	Ref.
DEHP (11 days × 500 mg/kg/d)	BALB/c mice; lung epithelial cells (n = N/S)	miR-146b-5p	↑	MicroRNA sequencing, qRT-PCR	ELISA, Bioinformatic analysis, miRanda, RNAhybrid, DAVID, GO, KEGG	PI3K/AKT pathway	[115]

Note: ^a^ refers to mean level detected in human studies; ^b^ refers to dose used in in vivo/in vitro studies ^c^ refers to miRNA detection; not specified (N/S); ↑, upregulation.

## Data Availability

No new data were created or analysed in this study. Data sharing is not applicable to this article.

## Abbreviations

The following abbreviations are used in this manuscript:

EDCs	Endocrine-disrupting chemicals
PCPs	Personal care products
LMWP	Low-molecular-weight phthalates
HMWP	High-molecular-weight phthalates
DEHP	Di-2-ethylhexyl-phthalate
DBP	Di-n-butyl phthalate
BzBP	Benzyl butyl phthalate
DiBP	Di-isobutyl phthalate
EV-miRNA	Extracellular vesicle microRNA
pri-miRNA	Primary-miRNA
pre-miRNA	Precursor-miRNA
miRISC	miRNA-induced silencing complex
miRNAs	MicroRNAs
mRNA	Messenger RNA
MBzP	Monobenzyl phthalate
MEP	Monoethyl phthalate
MCOMOP	Mono(3-carboxypropyl) phthalate
MCOMHP	Mono(3-carboxy-2-hydroxypropyl) phthalate
MECPP	Mono(2-ethyl-5-carboxypentyl) phthalate
MEHP	Mono(2-ethylhexyl) phthalate
MHBP	Mono-3-hydroxybutyl phthalate
MEHHP	Mono-2-ethyl-5-hydroxyhexyl phthalate
MEOHP	Mono-2-ethyl-5-oxohexyl phthalate
cAMP	Cyclic adenosine monophosphate
PKA	Protein kinase A
PPARG	Peroxisome proliferator-activated receptor gamma
TGF-β	Transforming growth factor-beta
HTR 8/Svneo	Human trophoblast cell line
PI3K	Phosphoinositide 3-kinase
AKT	Protein kinase B
PTEN	Phosphatase and tensin homolog
SOD	Superoxide dismutase
BCL-2	B-cell lymphoma 2
BAX	Bcl-2-associated X protein
RNO	Rat
Star	Steroidogenic acute regulatory protein
mTOR	Mechanistic target of rapamycin
FoxO	Forkhead box O transcription factor
Hippo	Hippo signalling pathway
KEGG	Kyoto Encyclopaedia of Genes and Genomes
IPAGOSP	Ingenuity Pathway AnalysisGene ontologySeminal plasma
MBP	Mono-n-butyl phthalate
SCs	Sertoli cells
qRT-PCR	Quantitative reverse transcription polymerase chain reaction
Rasd1	Ras-related protein 1
DNMT3b	DNA methyltransferase 3 beta
GC	Germ cells
PDK4	Pyruvate dehydrogenase lipoamide kinase isozyme 4
ER(+)	Oestrogen receptor-positive
PCNA	Proliferating cell nuclear antigen
FZD7	Frizzled class receptor 7
Wnt	Wingless-related integration site
FXYD6	FXYD domain containing ion transport regulator 6
OSCC	Oral squamous cell carcinoma
sEV	Small extracellular vesicle
ALT	Alanine aminotransferase
AST	Aspartate aminotransferase
iNOS	Inducible nitric oxide synthase
Notch1	Notch receptor 1
BaP	Benzo[a]pyrene
DiDP	Diisodecyl phthalate
elovl6	Elongation of very long chain fatty acids protein 6
abhd6a	Alpha/beta hydrolase domain-containing protein 6A
cel	Cysteinyl leukotriene receptor 1
pla2g12b	Phospholipase A2 group 12B
pxr	Pregnane X receptor
pla2g6	Phospholipase A2 group 6
SOCS1	Suppressor of cytokine signalling 1
STAT3	Signal transducer and activator of transcription 3
SIRT1	Sirtuin 1
Nampt	Nicotinamide phosphoribosyltransferase
IRS1	Insulin receptor substrate 1
GLUT4	Glucose transporter type 4
lncRNA	Long non-coding RNA
Keap1	Kelch-like ECH-associated protein 1
Nrf2	Nuclear factor erythroid 2-related factor 2
GAS5	Growth arrest-specific 5
PCSK9	Proprotein convertase subtilisin/kexin type 9
VSMC	Vascular smooth muscle cell
MCP-1	Monocyte chemoattractant protein-1
SP1	Specificity protein 1
MYOCD	Myocardin
THP-1	Human monocytic cell line
hpf	Hours post-fertilization
PRPF3	Pre-mRNA processing factor 3
UTR	Untranslated region
OVA	Ovalbumin
Th1	T-helper 1 cells
Th2	T-helper 2 cells
Treg	Regulatory T cells
SD rats	Sprague Dawley rats
C-MYC	Cellular myelocytomatosis oncogene
AML	Acute myeloid leukaemia

## References

[1] Saravanabhavan G., Murray J. (2012). Human biological monitoring of diisononyl phthalate and diisodecyl phthalate: A review. J. Environ. Public Health.

[2] Bu S., Wang Y., Wang H., Wang F., Tan Y. (2020). Analysis of global commonly-used phthalates and non-dietary exposure assessment in indoor environment. Build. Environ..

[3] Gao D., Li Z., Wang H., Liang H. (2018). An overview of phthalate acid ester pollution in China over the last decade: Environmental occurrence and human exposure. Sci. Total Environ..

[4] Wang Y., Zhu H., Kannan K. (2019). A Review of Biomonitoring of Phthalate Exposures. Toxics.

[5] Li C., Xu J., Chen D., Xiao Y. (2016). Detection of phthalates migration from disposable tablewares to drinking water using hexafluoroisopropanol-induced catanionic surfactant coacervate extraction. J. Pharm. Anal..

[6] Tuan Tran H., Lin C., Bui X.-T., Ky Nguyen M., Dan Thanh Cao N., Mukhtar H., Giang Hoang H., Varjani S., Hao Ngo H., Nghiem L.D. (2022). Phthalates in the environment: Characteristics, fate and transport, and advanced wastewater treatment technologies. Bioresour. Technol..

[7] Zhang Y.J., Guo J.L., Xue J.C., Bai C.L., Guo Y. (2021). Phthalate metabolites: Characterization, toxicities, global distribution, and exposure assessment. Environ. Pollut..

[8] National Research Council (2008). National Research Council Committee on the Health Risks of Phthalates.

[9] Cao X.L. (2010). Phthalate Esters in Foods: Sources, Occurrence, and Analytical Methods. Compr. Rev. Food Sci. Food Saf..

[10] Koch H.M., Bolt H.M., Angerer J. (2004). Di(2-ethylhexyl)phthalate (DEHP) metabolites in human urine and serum after a single oral dose of deuterium-labelled DEHP. Arch. Toxicol..

[11] Koch H.M., Bolt H.M., Preuss R., Angerer J. (2005). New metabolites of di(2-ethylhexyl)phthalate (DEHP) in human urine and serum after single oral doses of deuterium-labelled DEHP. Arch. Toxicol..

[12] Frederiksen H., Skakkebaek N.E., Andersson A.M. (2007). Metabolism of phthalates in humans. Mol. Nutr. Food Res..

[13] Silano V., Barat Baviera J.M., Bolognesi C., Chesson A., Cocconcelli P.S., Crebelli R., Gott D.M., Grob K., Lampi E., Mortensen A. (2019). Update of the risk assessment of di-butylphthalate (DBP), butyl-benzyl-phthalate (BBP), bis(2-ethylhexyl)phthalate (DEHP), di-isononylphthalate (DINP) and di-isodecylphthalate (DIDP) for use in food contact materials. Efsa J..

[14] Gore A.C., Chappell V.A., Fenton S.E., Flaws J.A., Nadal A., Prins G.S., Toppari J., Zoeller R.T. (2015). EDC-2: The Endocrine Society’s Second Scientific Statement on Endocrine-Disrupting Chemicals. Endocr. Rev..

[15] Eales J., Bethel A., Galloway T., Hopkinson P., Morrissey K., Short R.E., Garside R. (2022). Human health impacts of exposure to phthalate plasticizers: An overview of reviews. Environ. Int..

[16] Wang Y., Qian H. (2021). Phthalates and Their Impacts on Human Health. Healthcare.

[17] Publications Office of the European Union (2018). Commission Regulation (EU) 2018/2005.

[18] Bartel D.P. (2004). MicroRNAs: Genomics, biogenesis, mechanism, and function. Cell.

[19] Rodriguez A., Griffiths-Jones S., Ashurst J.L., Bradley A. (2004). Identification of mammalian microRNA host genes and transcription units. Genome Res..

[20] Bhaskaran M., Mohan M. (2014). MicroRNAs: History, biogenesis, and their evolving role in animal development and disease. Vet. Pathol..

[21] O’Brien J., Hayder H., Zayed Y., Peng C. (2018). Overview of MicroRNA Biogenesis, Mechanisms of Actions, and Circulation. Front. Endocrinol..

[22] Ruby J.G., Jan C.H., Bartel D.P. (2007). Intronic microRNA precursors that bypass Drosha processing. Nature.

[23] Cheloufi S., Dos Santos C.O., Chong M.M., Hannon G.J. (2010). A dicer-independent miRNA biogenesis pathway that requires Ago catalysis. Nature.

[24] Correia de Sousa M., Gjorgjieva M., Dolicka D., Sobolewski C., Foti M. (2019). Deciphering miRNAs’ Action through miRNA Editing. Int. J. Mol. Sci..

[25] Lytle J.R., Yario T.A., Steitz J.A. (2007). Target mRNAs are repressed as efficiently by microRNA-binding sites in the 5′ UTR as in the 3′ UTR. Proc. Natl. Acad. Sci. USA.

[26] Dharap A., Pokrzywa C., Murali S., Pandi G., Vemuganti R. (2013). MicroRNA miR-324-3p induces promoter-mediated expression of RelA gene. PLoS ONE.

[27] Wang J., Chen J., Sen S. (2016). MicroRNA as Biomarkers and Diagnostics. J. Cell. Physiol..

[28] Mori M.A., Ludwig R.G., Garcia-Martin R., Brandão B.B., Kahn C.R. (2019). Extracellular miRNAs: From Biomarkers to Mediators of Physiology and Disease. Cell Metab..

[29] Kosaka N., Iguchi H., Yoshioka Y., Takeshita F., Matsuki Y., Ochiya T. (2010). Secretory mechanisms and intercellular transfer of microRNAs in living cells. J. Biol. Chem..

[30] Gjorgjieva M., Sobolewski C., Dolicka D., Correia de Sousa M., Foti M. (2019). miRNAs and NAFLD: From pathophysiology to therapy. Gut.

[31] Li Y., Baumert B.O., Costello E., Chen J.C., Rock S., Stratakis N., Goodrich J.A., Zhao Y., Eckel S.P., Walker D.I. (2024). Per- and polyfluoroalkyl substances, polychlorinated biphenyls, organochlorine pesticides, and polybrominated diphenyl ethers and dysregulation of MicroRNA expression in humans and animals—A systematic review. Environ. Res..

[32] Mozzoni P., Poli D., Pinelli S., Tagliaferri S., Corradi M., Cavallo D., Ursini C.L., Pigini D. (2023). Benzene Exposure and MicroRNAs Expression: In Vitro, In Vivo and Human Findings. Int. J. Environ. Res. Public Health.

[33] Tumolo M.R., Panico A., De Donno A., Mincarone P., Leo C.G., Guarino R., Bagordo F., Serio F., Idolo A., Grassi T. (2022). The expression of microRNAs and exposure to environmental contaminants related to human health: A review. Int. J. Environ. Health Res..

[34] Arrigo F., Impellitteri F., Piccione G., Faggio C. (2023). Phthalates and their effects on human health: Focus on erythrocytes and the reproductive system. Comp. Biochem. Physiol. C Toxicol. Pharmacol..

[35] Hlisníková H., Petrovičová I., Kolena B., Šidlovská M., Sirotkin A. (2020). Effects and Mechanisms of Phthalates’ Action on Reproductive Processes and Reproductive Health: A Literature Review. Int. J. Environ. Res. Public Health.

[36] Zhong J., Baccarelli A.A., Mansur A., Adir M., Nahum R., Hauser R., Bollati V., Racowsky C., Machtinger R. (2019). Maternal Phthalate and Personal Care Products Exposure Alters Extracellular Placental miRNA Profile in Twin Pregnancies. Reprod. Sci..

[37] LaRocca J., Binder A.M., McElrath T.F., Michels K.B. (2016). First-Trimester Urine Concentrations of Phthalate Metabolites and Phenols and Placenta miRNA Expression in a Cohort of U.S. Women. Environ. Health Perspect..

[38] Gu X., Liu H., Luo W., Wang X., Wang H., Li L. (2022). Di-2-ethylhexyl phthalate-induced miR-155-5p promoted lipid metabolism via inhibiting cAMP/PKA signaling pathway in human trophoblastic HTR-8/Svneo cells. Reprod. Toxicol..

[39] Meruvu S., Zhang J., Choudhury M. (2016). Mono-(2-ethylhexyl) Phthalate Increases Oxidative Stress Responsive miRNAs in First Trimester Placental Cell Line HTR8/SVneo. Chem. Res. Toxicol..

[40] Meruvu S., Zhang J., Bedi Y.S., Choudhury M. (2016). Mono-(2-ethylhexyl) phthalate induces apoptosis through miR-16 in human first trimester placental cell line HTR-8/SVneo. Toxicol. Vitr..

[41] Zota A.R., Geller R.J., VanNoy B.N., Marfori C.Q., Tabbara S., Hu L.Y., Baccarelli A.A., Moawad G.N. (2020). Phthalate Exposures and MicroRNA Expression in Uterine Fibroids: The FORGE Study. Epigenet. Insights.

[42] Barnett-Itzhaki Z., Knapp S., Avraham C., Racowsky C., Hauser R., Bollati V., Baccarelli A.A., Machtinger R. (2021). Association between follicular fluid phthalate concentrations and extracellular vesicle microRNAs expression. Hum. Reprod..

[43] Martinez R.M., Hauser R., Liang L., Mansur A., Adir M., Dioni L., Racowsky C., Bollati V., Baccarelli A.A., Machtinger R. (2019). Urinary concentrations of phenols and phthalate metabolites reflect extracellular vesicle microRNA expression in follicular fluid. Environ. Int..

[44] Liu J., Wang W., Zhu J., Li Y., Luo L., Huang Y., Zhang W. (2018). Di(2-ethylhexyl) phthalate (DEHP) influences follicular development in mice between the weaning period and maturity by interfering with ovarian development factors and microRNAs. Environ. Toxicol..

[45] Gonsioroski A.V., Aquino A.M., Alonso-Costa L.G., Barbisan L.F., Scarano W.R., Flaws J.A. (2022). Multigenerational effects of an environmentally relevant phthalate mixture on reproductive parameters and ovarian miRNA expression in female rats. Toxicol. Sci..

[46] Zhang J.N., Zhang R.Q., Liu J.C., Li L., Shen W., Sun X.F. (2019). Di (2-ethylhexyl) Phthalate Exposure Impairs the microRNAs Expression Profile During Primordial Follicle Assembly. Front. Endocrinol..

[47] Scarano W.R., Bedrat A., Alonso-Costa L.G., Aquino A.M., Fantinatti B., Justulin L.A., Barbisan L.F., Freire P.P., Flaws J.A., Bernardo L. (2019). Exposure to an environmentally relevant phthalate mixture during prostate development induces microRNA upregulation and transcriptome modulation in rats. Toxicol. Sci..

[48] Oluwayiose O.A., Houle E., Whitcomb B.W., Suvorov A., Rahil T., Sites C.K., Krawetz S.A., Visconti P.E., Pilsner J.R. (2023). Urinary phthalate metabolites and small non-coding RNAs from seminal plasma extracellular vesicles among men undergoing infertility treatment. Environ. Pollut..

[49] Helmy H.S., Senousy M.A., El-Sahar A.E., Sayed R.H., Saad M.A., Elbaz E.M. (2020). Aberrations of miR-126-3p, miR-181a and sirtuin1 network mediate Di-(2-ethylhexyl) phthalate-induced testicular damage in rats: The protective role of hesperidin. Toxicology.

[50] Tang M., Zhang L., Zhu Z., Li R., Wang S., Wang W., Qin Z., Zhang W. (2020). Overexpression of miR-506-3p Aggravates DBP-Induced Testicular Oxidative Stress in Rats by Downregulating ANXA5 via Nrf2/HO-1 Signaling Pathway. Oxid. Med. Cell. Longev..

[51] Yuan B., Wu W., Zhang H., Gu H., Guo D., Jiang J., Wang X. (2019). Adenomatous polyposis coli as a predictor of environmental chemical-induced transgenerational effects related to male infertility. J. Biochem. Mol. Toxicol..

[52] Buñay J., Larriba E., Moreno R.D., Del Mazo J. (2017). Chronic low-dose exposure to a mixture of environmental endocrine disruptors induces microRNAs/isomiRs deregulation in mouse concomitant with intratesticular estradiol reduction. Sci. Rep..

[53] Yin X., Ma T., Han R., Ding J., Zhang H., Han X., Li D. (2018). MiR-301b-3p/3584-5p enhances low-dose mono-n-butyl phthalate (MBP)-induced proliferation by targeting Rasd1 in Sertoli cells. Toxicol. In Vitro.

[54] Li R., Xing Q.W., Wu X.L., Zhang L., Tang M., Tang J.Y., Wang J.Z., Han P., Wang S.Q., Wang W. (2019). Di-n-butyl phthalate epigenetically induces reproductive toxicity via the PTEN/AKT pathway. Cell Death Dis..

[55] Štefánik P., Michalec J., Morová M., Olexová L., Kršková L. (2022). Prenatal and perinatal phthalate exposure is associated with sex-dependent changes in hippocampal miR-15b-5p and miR-34a-5p expression and changes in testicular morphology in rat offspring. Arh. Hig. Rada Toksikol..

[56] Lu H., Zhang C., Hu Y., Qin H., Gu A., Li Y., Zhang L., Li Z., Wang Y. (2016). miRNA-200c mediates mono-butyl phthalate-disrupted steroidogenesis by targeting vimentin in Leydig tumor cells and murine adrenocortical tumor cells. Toxicol. Lett..

[57] Vashukova E.S., Glotov A.S., Fedotov P.V., Efimova O.A., Pakin V.S., Mozgovaya E.V., Pendina A.A., Tikhonov A.V., Koltsova A.S., Baranov V.S. (2016). Placental microRNA expression in pregnancies complicated by superimposed pre-eclampsia on chronic hypertension. Mol. Med. Rep..

[58] Ishibashi O., Ohkuchi A., Ali M.M., Kurashina R., Luo S.S., Ishikawa T., Takizawa T., Hirashima C., Takahashi K., Migita M. (2012). Hydroxysteroid (17-β) dehydrogenase 1 is dysregulated by miR-210 and miR-518c that are aberrantly expressed in preeclamptic placentas: A novel marker for predicting preeclampsia. Hypertension.

[59] Wang J., Zhang Y., Zhang W., Jin Y., Dai J. (2012). Association of perfluorooctanoic acid with HDL cholesterol and circulating miR-26b and miR-199-3p in workers of a fluorochemical plant and nearby residents. Environ. Sci. Technol..

[60] Fu Z., Zhao F., Chen K., Xu J., Li P., Xia D., Wu Y. (2017). Association between urinary phthalate metabolites and risk of breast cancer and uterine leiomyoma. Reprod. Toxicol..

[61] Yang Y., Bai X., Lu J., Zou R., Ding R., Hua X. (2023). Assessment of five typical environmental endocrine disruptors and thyroid cancer risk: A meta-analysis. Front. Endocrinol..

[62] Chuang S.-C., Chen H.-C., Sun C.-W., Chen Y.-A., Wang Y.-H., Chiang C.-J., Chen C.-C., Wang S.-L., Chen C.-J., Hsiung C.A. (2020). Phthalate exposure and prostate cancer in a population-based nested case-control study. Environ. Res..

[63] Corti M., Lorenzetti S., Ubaldi A., Zilli R., Marcoccia D. (2022). Endocrine Disruptors and Prostate Cancer. Int. J. Mol. Sci..

[64] Guo T., Meng X., Liu X., Wang J., Yan S., Zhang X., Wang M., Ren S., Huang Y. (2023). Associations of phthalates with prostate cancer among the US population. Reprod. Toxicol..

[65] Kim J.H. (2019). Di(2-ethylhexyl) phthalate promotes lung cancer cell line A549 progression via Wnt/β-catenin signaling. J. Toxicol. Sci..

[66] Hsu Y.L., Hung J.Y., Tsai E.M., Wu C.Y., Ho Y.W., Jian S.F., Yen M.C., Chang W.A., Hou M.F., Kuo P.L. (2015). Benzyl butyl phthalate increases the chemoresistance to doxorubicin/cyclophosphamide by increasing breast cancer-associated dendritic cell-derived CXCL1/GROα and S100A8/A9. Oncol. Rep..

[67] Wang Y.C., Tsai C.F., Chuang H.L., Chang Y.C., Chen H.S., Lee J.N., Tsai E.M. (2016). Benzyl butyl phthalate promotes breast cancer stem cell expansion via SPHK1/S1P/S1PR3 signaling. Oncotarget.

[68] Zhu M., Wu J., Ma X., Huang C., Wu R., Zhu W., Li X., Liang Z., Deng F., Zhu J. (2019). Butyl benzyl phthalate promotes prostate cancer cell proliferation through miR-34a downregulation. Toxicol. In Vitro.

[69] Cavalca A.M.B., Aquino A.M., Mosele F.C., Justulin L.A., Delella F.K., Flaws J.A., Scarano W.R. (2022). Effects of a phthalate metabolite mixture on both normal and tumoral human prostate cells. Environ. Toxicol..

[70] Wu J., Jiang Y., Cao W., Li X., Xie C., Geng S., Zhu M., Liang Z., Zhu J., Zhu W. (2018). miR-19 targeting of PTEN mediates butyl benzyl phthalate-induced proliferation in both ER(+) and ER(-) breast cancer cells. Toxicol. Lett..

[71] Cao W., Lu X., Zhong C., Wu J. (2023). Sulforaphane Suppresses MCF-7 Breast Cancer Cells Growth via miR-19/PTEN Axis to Antagonize the Effect of Butyl Benzyl Phthalate. Nutr. Cancer.

[72] Wang M., Qiu Y., Zhang R., Gao L., Wang X., Bi L., Wang Y. (2019). MEHP promotes the proliferation of oral cancer cells via down regulation of miR-27b-5p and miR-372-5p. Toxicol. In Vitro.

[73] Liu B., Chen W., Cao G., Dong Z., Xu J., Luo T., Zhang S. (2017). MicroRNA-27b inhibits cell proliferation in oral squamous cell carcinoma by targeting FZD7 and Wnt signaling pathway. Arch. Oral Biol..

[74] Xu S.Y., Xu P.F., Gao T.T. (2018). MiR-372-3p inhibits the growth and metastasis of osteosarcoma cells by targeting FXYD6. Eur. Rev. Med. Pharmacol. Sci..

[75] Qin Y., Zhang J., Avellán-Llaguno R.D., Zhang X., Huang Q. (2021). DEHP-elicited small extracellular vesicles miR-26a-5p promoted metastasis in nearby normal A549 cells. Environ. Pollut..

[76] Duan X.L., Ma C.C., Hua J., Xiao T.W., Luan J. (2020). Benzyl butyl phthalate (BBP) triggers the malignancy of acute myeloid leukemia cells via upregulation of PDK4. Toxicol. In Vitro.

[77] Cui L., Cheng Z., Liu Y., Dai Y., Pang Y., Jiao Y., Ke X., Cui W., Zhang Q., Shi J. (2020). Overexpression of PDK2 and PDK3 reflects poor prognosis in acute myeloid leukemia. Cancer Gene Ther..

[78] Weng Y., Shen Y., He Y., Pan X., Xu J., Jiang Y., Zhang Q., Wang S., Kong F., Zhao S. (2018). The miR-15b-5p/PDK4 axis regulates osteosarcoma proliferation through modulation of the Warburg effect. Biochem. Biophys. Res. Commun..

[79] Liu F., Chen Q., Chen F., Wang J., Gong R., He B. (2019). The lncRNA ENST00000608794 acts as a competing endogenous RNA to regulate PDK4 expression by sponging miR-15b-5p in dexamethasone induced steatosis. Biochim. Biophys. Acta Mol. Cell Biol. Lipids.

[80] Chung G.E., Yoon J.H., Myung S.J., Lee J.H., Lee S.H., Lee S.M., Kim S.J., Hwang S.Y., Lee H.S., Kim C.Y. (2010). High expression of microRNA-15b predicts a low risk of tumor recurrence following curative resection of hepatocellular carcinoma. Oncol. Rep..

[81] Grün F. (2010). Obesogens. Curr. Opin. Endocrinol. Diabetes Obes..

[82] Grün F., Blumberg B. (2009). Minireview: The case for obesogens. Mol. Endocrinol..

[83] Grün F., Blumberg B. (2007). Perturbed nuclear receptor signaling by environmental obesogens as emerging factors in the obesity crisis. Rev. Endocr. Metab. Disord..

[84] Grün F., Blumberg B. (2009). Endocrine disrupters as obesogens. Mol. Cell. Endocrinol..

[85] Grün F., Blumberg B. (2006). Environmental obesogens: Organotins and endocrine disruption via nuclear receptor signaling. Endocrinology.

[86] Tian M., Zhang X., Liu L., Martin F.L., Wang H., Zhang J., Huang Q., Wang X., Shen H. (2019). Phthalate side-chain structures and hydrolysis metabolism associated with steroidogenic effects in MLTC-1 Leydig cells. Toxicol. Lett..

[87] Lee S., Lee H.A., Park B., Han H., Hong Y.S., Ha E.H., Park H. (2023). Prospective association between phthalate exposure in childhood and liver function in adolescence: The Ewha Birth and Growth Cohort Study. Environ. Health.

[88] Radke E.G., Galizia A., Thayer K.A., Cooper G.S. (2019). Phthalate exposure and metabolic effects: A systematic review of the human epidemiological evidence. Environ. Int..

[89] Mariana M., Cairrao E. (2020). Phthalates Implications in the Cardiovascular System. J. Cardiovasc. Dev. Dis..

[90] Chen W., Liu Y., Chen J., Song Y., You M., Yang G. (2022). Long-term co-exposure DBP and BaP causes imbalance in liver macrophages polarization via activation of Notch signaling regulated by miR-34a-5p in rats. Chem. Biol. Interact..

[91] Chorley B.N., Carswell G.K., Nelson G., Bhat V.S., Wood C.E. (2020). Early microRNA indicators of PPARα pathway activation in the liver. Toxicol. Rep..

[92] Cocci P., Mosconi G., Palermo F.A. (2019). Changes in expression of microRNA potentially targeting key regulators of lipid metabolism in primary gilthead sea bream hepatocytes exposed to phthalates or flame retardants. Aquat. Toxicol..

[93] Liu Y., Chen W., Chen J., Ma Y., Cen Y., Wang S., He X., You M., Yang G. (2021). miR-122-5p regulates hepatocytes damage caused by BaP and DBP co-exposure through SOCS1/STAT3 signaling in vitro. Ecotoxicol. Environ. Saf..

[94] Meruvu S., Zhang J., Choudhury M. (2021). Butyl Benzyl Phthalate Promotes Adipogenesis in 3T3-L1 Cells via the miRNA-34a-5p Signaling Pathway in the Absence of Exogenous Adipogenic Stimuli. Chem. Res. Toxicol..

[95] Zhang J., Choudhury M. (2021). Benzyl Butyl Phthalate Induced Early lncRNA H19 Regulation in C3H10T1/2 Stem Cell Line. Chem. Res. Toxicol..

[96] Wei J., Hao Q., Chen C., Li J., Han X., Lei Z., Wang T., Wang Y., You X., Chen X. (2020). Epigenetic repression of miR-17 contributed to di(2-ethylhexyl) phthalate-triggered insulin resistance by targeting Keap1-Nrf2/miR-200a axis in skeletal muscle. Theranostics.

[97] Liu C., Qin Q., Xu J., Li X., Cong H. (2022). Phthalate promotes atherosclerosis through interacting with long-non coding RNA and induces macrophage foam cell formation and vascular smooth muscle damage. Chemosphere.

[98] Wen Y., Kong Y., Cao G., Xu Y., Zhang C., Zhang J., Xiao P., Wang Y. (2022). Di-n-butyl phthalate regulates vascular smooth muscle cells phenotypic switching by MiR-139-5p-MYOCD pathways. Toxicology.

[99] Kong Y., Wen Y., Cao G., Xu Y., Zhang C., Tang C., Zhang J., Wang Y. (2022). Di-n-butyl phthalate promotes monocyte recruitment via miR-137-3p-SP1-MCP-1 pathway. Ecotoxicol. Environ. Saf..

[100] Wang Y., Wen Y., Xiao P., Sun J., Chen M., Gu C., Kong Y., Gu A., Zhang J., Wang Y. (2020). Di-n-butyl phthalate promotes lipid accumulation via the miR200c-5p-ABCA1 pathway in THP-1 macrophages. Environ. Pollut..

[101] Mai E.H., Lei T., Li S.Q., Hu P.G., Xu T., Jia F.X., Zha Z.M., Zhang S.J., Ding F.H. (2019). MiR-34a affects hepatocyte proliferation during hepatocyte regeneration through regulating Notch/HIF-1α signaling pathway. Eur. Rev. Med. Pharmacol. Sci..

[102] Ortega F.J., Moreno-Navarrete J.M., Pardo G., Sabater M., Hummel M., Ferrer A., Rodriguez-Hermosa J.I., Ruiz B., Ricart W., Peral B. (2010). MiRNA expression profile of human subcutaneous adipose and during adipocyte differentiation. PLoS ONE.

[103] Wylie A.C., Short S.J. (2023). Environmental Toxicants and the Developing Brain. Biol. Psychiatry.

[104] Ferguson K.K., Loch-Caruso R., Meeker J.D. (2012). Exploration of Oxidative Stress and Inflammatory Markers in Relation to Urinary Phthalate Metabolites: NHANES 1999–2006. Environ. Sci. Technol..

[105] Ferguson K.K., Chen Y.H., VanderWeele T.J., McElrath T.F., Meeker J.D., Mukherjee B. (2017). Mediation of the Relationship between Maternal Phthalate Exposure and Preterm Birth by Oxidative Stress with Repeated Measurements across Pregnancy. Environ. Health Perspect..

[106] Ejaredar M., Nyanza E.C., Ten Eycke K., Dewey D. (2015). Phthalate exposure and childrens neurodevelopment: A systematic review. Environ. Res..

[107] Lee D.-W., Kim M.-S., Lim Y.-H., Lee N., Hong Y.-C. (2018). Prenatal and postnatal exposure to di-(2-ethylhexyl) phthalate and neurodevelopmental outcomes: A systematic review and meta-analysis. Environ. Res..

[108] Radke E.G., Braun J.M., Nachman R.M., Cooper G.S. (2020). Phthalate exposure and neurodevelopment: A systematic review and meta-analysis of human epidemiological evidence. Environ. Int..

[109] Niermann S., Rattan S., Brehm E., Flaws J.A. (2015). Prenatal exposure to di-(2-ethylhexyl) phthalate (DEHP) affects reproductive outcomes in female mice. Reprod. Toxicol..

[110] Luu B.E., Green S.R., Childers C.L., Holahan M.R., Storey K.B. (2017). The roles of hippocampal microRNAs in response to acute postnatal exposure to di(2-ethylhexyl) phthalate in female and male rats. Neurotoxicology.

[111] Qiu F., He S., Zhang Z., Dai S., Wang J., Liu N., Li Z., Hu X., Xiang S., Wei C. (2023). MiR-93 alleviates DEHP plasticizer-induced neurotoxicity by negatively regulating TNFAIP1 and inhibiting ubiquitin-mediated degradation of CK2β. Food Chem. Toxicol..

[112] Du Y.T., Yu J.J., Huang G., Zhang K., Abdelhafez H.E.H., Yin X.H., Qiao J.K., Guo J.F. (2021). Regulation of TRAF6 by MicroRNA-146a in Zebrafish Embryos after Exposure to Di(2-Ethylhexyl) Phthalate at Different Concentrations. Chem. Res. Toxicol..

[113] Yu J., Huang G., Gong Q., Zhang K., Abdelhafez H., Du Y., Guo J. (2023). MicroRNA-375 Mediated Regulation on Pre-mRNA Processing Factor 3 in Zebrafish Embryos Exposed to Di-(2-ethylhexyl)phthalate at Low Concentrations. Chem. Res. Toxicol..

[114] Bølling A.K., Sripada K., Becher R., Bekö G. (2020). Phthalate exposure and allergic diseases: Review of epidemiological and experimental evidence. Environ. Int..

[115] Liu P., Quan X., Zhang Q., Chen Y., Wang X., Xu C., Li N. (2023). Multi-omics reveals the mechanisms of DEHP driven pulmonary toxicity in ovalbumin-sensitized mice. Ecotoxicol. Environ. Saf..

[116] Hunt E.A., Broyles D., Head T., Deo S.K. (2015). MicroRNA Detection: Current Technology and Research Strategies. Annu. Rev. Anal. Chem..

[117] Chua C.E.L., Tang B.L. (2019). miR-34a in Neurophysiology and Neuropathology. J. Mol. Neurosci..

[118] Zhang L., Liao Y., Tang L. (2019). MicroRNA-34 family: A potential tumor suppressor and therapeutic candidate in cancer. J. Exp. Clin. Cancer Res..

[119] Li Q., Zhang Q. (2023). MiR-34a and endothelial biology. Life Sci..

[120] Hart M., Walch-Rückheim B., Friedmann K.S., Rheinheimer S., Tänzer T., Glombitza B., Sester M., Lenhof H.P., Hoth M., Schwarz E.C. (2019). miR-34a: A new player in the regulation of T cell function by modulation of NF-κB signaling. Cell Death Dis..

[121] Doridot L., Houry D., Gaillard H., Chelbi S.T., Barbaux S., Vaiman D. (2014). miR-34a expression, epigenetic regulation, and function in human placental diseases. Epigenetics.

[122] Gagnon J.D., Kageyama R., Shehata H.M., Fassett M.S., Mar D.J., Wigton E.J., Johansson K., Litterman A.J., Odorizzi P., Simeonov D. (2019). miR-15/16 Restrain Memory T Cell Differentiation, Cell Cycle, and Survival. Cell Rep..

[123] Xia H., Qi Y., Ng S.S., Chen X., Chen S., Fang M., Li D., Zhao Y., Ge R., Li G. (2009). MicroRNA-15b regulates cell cycle progression by targeting cyclins in glioma cells. Biochem. Biophys. Res. Commun..

[124] Sun G., Yan S., Shi L., Wan Z., Jiang N., Li M., Guo J. (2015). Decreased Expression of miR-15b in Human Gliomas is Associated with Poor Prognosis. Cancer Biother. Radiopharm..

[125] Qi L.Q., Sun B., Yang B.B., Lu S. (2020). MiR-15b facilitates breast cancer progression via repressing tumor suppressor PAQR3. Eur. Rev. Med. Pharmacol. Sci..

[126] Martino E., D’Onofrio N., Balestrieri A., Mele L., Sardu C., Marfella R., Campanile G., Balestrieri M.L. (2023). MiR-15b-5p and PCSK9 inhibition reduces lipopolysaccharide-induced endothelial dysfunction by targeting SIRT4. Cell Mol. Biol. Lett..

[127] Li W., Cui Y., Wang D., Wang Y., Wang L. (2019). MiR-141-3p functions as a tumor suppressor through directly targeting ZFR in non-small cell lung cancer. Biochem. Biophys. Res. Commun..

[128] Bao J., Li X., Li Y., Huang C., Meng X., Li J. (2019). MicroRNA-141-5p Acts as a Tumor Suppressor via Targeting RAB32 in Chronic Myeloid Leukemia. Front. Pharmacol..

[129] Abedi N., Mohammadi-Yeganeh S., Koochaki A., Karami F., Paryan M. (2015). miR-141 as potential suppressor of β-catenin in breast cancer. Tumour Biol..

[130] Wang M., Hu M., Li Z., Qian D., Wang B., Liu D.X. (2017). miR-141-3p functions as a tumor suppressor modulating activating transcription factor 5 in glioma. Biochem. Biophys. Res. Commun..

[131] Liu S., Wen C. (2022). miR-141-3p promotes retinoblastoma progression via inhibiting sushi domain-containing protein 2. Bioengineered.

[132] Luo Q., Tian Y., Qu G., Huang K., Hu P., Li L., Luo S. (2023). MiR-141-3p promotes hypoxia-induced autophagy in human placental trophoblast cells. Reprod. Biol..

[133] Yang G., Lu Z., Meng F., Wan Y., Zhang L., Xu Q., Wang Z. (2022). Circulating miR-141 as a potential biomarker for diagnosis, prognosis and therapeutic targets in gallbladder cancer. Sci. Rep..

[134] Li Y., Gu F., Lin X. (2020). The role of miR-141/ Sirt1 in colon cancer. J. Buon.

[135] Jafari M., Ghadami E., Dadkhah T., Akhavan-Niaki H. (2019). PI3k/AKT signaling pathway: Erythropoiesis and beyond. J. Cell. Physiol..

[136] Karami Fath M., Ebrahimi M., Nourbakhsh E., Zia Hazara A., Mirzaei A., Shafieyari S., Salehi A., Hoseinzadeh M., Payandeh Z., Barati G. (2022). PI3K/Akt/mTOR signaling pathway in cancer stem cells. Pathol. Res. Pract..

[137] Liu L., Yan M., Yang R., Qin X., Chen L., Li L., Si J., Li X., Ma K. (2021). Adiponectin Attenuates Lipopolysaccharide-induced Apoptosis by Regulating the Cx43/PI3K/AKT Pathway. Front. Pharmacol..

[138] Xu Y., Sui L., Qiu B., Yin X., Liu J., Zhang X. (2019). ANXA4 promotes trophoblast invasion via the PI3K/Akt/eNOS pathway in preeclampsia. Am. J. Physiol. Cell Physiol..

[139] Elmore S. (2007). Apoptosis: A review of programmed cell death. Toxicol. Pathol..

[140] Peng D., Fu M., Wang M., Wei Y., Wei X. (2022). Targeting TGF-β signal transduction for fibrosis and cancer therapy. Mol. Cancer.

[141] Zhou Y., Xu J., Luo H., Meng X., Chen M., Zhu D. (2022). Wnt signaling pathway in cancer immunotherapy. Cancer Lett..

[142] Gao Y., Chen N., Fu Z., Zhang Q. (2023). Progress of Wnt Signaling Pathway in Osteoporosis. Biomolecules.

[143] Jang D.I., Lee A.H., Shin H.Y., Song H.R., Park J.H., Kang T.B., Lee S.R., Yang S.H. (2021). The Role of Tumor Necrosis Factor Alpha (TNF-α) in Autoimmune Disease and Current TNF-α Inhibitors in Therapeutics. Int. J. Mol. Sci..

[144] Akash M.S.H., Rehman K., Liaqat A. (2018). Tumor Necrosis Factor-Alpha: Role in Development of Insulin Resistance and Pathogenesis of Type 2 Diabetes Mellitus. J. Cell. Biochem..

[145] Mai S., Mushinski J.F. (2003). c-Myc-induced genomic instability. J. Environ. Pathol. Toxicol. Oncol..

[146] Chen C.Y., Chen J., He L., Stiles B.L. (2018). PTEN: Tumor Suppressor and Metabolic Regulator. Front. Endocrinol..

[147] Sawe N., Steinberg G., Zhao H. (2008). Dual roles of the MAPK/ERK1/2 cell signaling pathway after stroke. J. Neurosci. Res..

[148] Jiang Q., Chen X., Tian X., Zhang J., Xue S., Jiang Y., Liu T., Wang X., Sun Q., Hong Y. (2022). Tanshinone I inhibits doxorubicin-induced cardiotoxicity by regulating Nrf2 signaling pathway. Phytomedicine.

[149] Lin L., Wu Q., Lu F., Lei J., Zhou Y., Liu Y., Zhu N., Yu Y., Ning Z., She T. (2023). Nrf2 signaling pathway: Current status and potential therapeutic targetable role in human cancers. Front. Oncol..

[150] Wang L., Zhang X., Xiong X., Zhu H., Chen R., Zhang S., Chen G., Jian Z. (2022). Nrf2 Regulates Oxidative Stress and Its Role in Cerebral Ischemic Stroke. Antioxidants.

[151] Pritchard C.C., Kroh E., Wood B., Arroyo J.D., Dougherty K.J., Miyaji M.M., Tait J.F., Tewari M. (2012). Blood cell origin of circulating microRNAs: A cautionary note for cancer biomarker studies. Cancer Prev. Res..

[152] Razak S.R., Ueno K., Takayama N., Nariai N., Nagasaki M., Saito R., Koso H., Lai C.Y., Murakami M., Tsuji K. (2013). Profiling of microRNA in human and mouse ES and iPS cells reveals overlapping but distinct microRNA expression patterns. PLoS ONE.

